# Metal Sulfide S‐Scheme Homojunction for Photocatalytic Selective Phenylcarbinol Oxidation

**DOI:** 10.1002/advs.202400099

**Published:** 2024-02-28

**Authors:** Huijun Zhang, Yujie Gao, Sugang Meng, Zengrong Wang, Peixian Wang, Zhongliao Wang, Chengwei Qiu, Shifu Chen, Bo Weng, Yu‐Ming Zheng

**Affiliations:** ^1^ Key Laboratory of Green and Precise Synthetic Chemistry and Applications Ministry of Education College of Chemistry and Materials Science Huaibei Normal University Huaibei 235000 P. R. China; ^2^ cMACS Department of Microbial and Molecular Systems KU Leuven Celestijnenlaan 200F Leuven 3001 Belgium; ^3^ High Field Magnetic Laboratory Hefei Institutes of Physical Science Chinese Academy of Sciences Hefei 230031 P. R. China; ^4^ School of Chemistry and Chemical Engineering/State Key Laboratory Incubation Base for Green Processing of Chemical Engineering Shihezi University Shihezi 832003 P. R. China; ^5^ State Key Lab of Photocatalysis on Energy and Environment College of Chemistry Fuzhou University Fuzhou 350116 P. R. China; ^6^ CAS Key Laboratory of Urban Pollutant Conversion Institute of Urban Environment, Chinese Academy of Sciences 1799 Jimei Road Xiamen 361021 P. R. China; ^7^ University of Chinese Academy of Sciences 19A Yuquan Road Beijing 100049 P. R. China

**Keywords:** homojunction, metal sulfide, selective oxidation of phenylcarbinol, S‐scheme

## Abstract

Metal sulfide‐based homojunction photocatalysts are extensively explored with improved photocatalytic performance. However, the construction of metal sulfide‐based S‐scheme homojunction remains a challenge. Herein, the fabrication of 2D CdIn_2_S_4_ nanosheets coated 3D CdIn_2_S_4_ octahedra (referred to as 2D/3D n‐CIS/o‐CIS) S‐scheme homojunction photocatalyst is reported by simply adjustment of polyvinyl pyrrolidone amount during the solvothermal synthesis. The formation of S‐scheme homojunction within n‐CIS/o‐CIS is systematically investigated via a series of characterizations, which can generate an internal electric field to facilitate the separation and migration of photogenerated electron‐hole pairs. The 2D/3D n‐CIS/o‐CIS composite exhibits significantly improved photocatalytic activity and stability in the selective oxidation of phenylcarbinol (PhCH_2_OH) to benzaldehyde (PhCHO) when compared to pure n‐CIS and o‐CIS samples under visible light irradiation. It is hoped that this work can contribute novel insights into the development of metal sulfides S‐scheme homojunction photocatalysts for solar energy conversion.

## Graphical abstract

The 2D/3D CdIn_2_S_4_ S‐scheme homojunction photocatalysts are reported for the first time through the straightforward adjustment of PVP amount, which generates an internal electric field to facilitate the separation and migration of photogenerated charge carriers, thus significantly improving the photocatalytic activity and stability of the selective oxidation of phenylcarbinol to benzaldehyde.

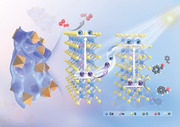

## 1 Introduction

Metal sulfide‐based semiconductor materials hold great promise in the realm of photocatalysis due to their intriguing properties, including narrow bandgaps, well‐aligned valence and conduction band positions, and their cost‐effective nature. Therefore, various metal sulfide semiconductors are recognized as important photocatalysts and widely utilized in the fields of water photolysis, CO_2_ reduction, and selective organic transformation.^[^ ^1^, ^2^, ^3^, ^4^, ^5^, ^6^ ^]^ Unfortunately, the practical applications of single metal sulfide photocatalysts are limited by the severe electron‐hole complexation and poor stability under light irradiation, thus leading to a low solar energy conversion efficiency. To overcome these challenges, extensive research endeavors have been dedicated to constructing different heterojunctions for forming multiple channels to accelerate the charge transfer process. For instance, the deposition of noble metals (e.g., Au, Pd, etc.) onto the surface of metal sulfides can form Schottky junctions to reduce charge recombination and offer abundant active sites for the reactions.^[^ ^7^, ^8^, ^9^, ^10^ ^]^ Integrating metal sulfides with other semiconductors, such as TiO_2_, ZnO, to create type II or Z‐scheme heterojunctions has also demonstrated significant potential for facilitating the migration of photoexcited charge carriers, consequently amplifying photocatalytic performance.^[^ ^11^, ^12^, ^13^ ^]^ However, the metal sulfide‐based heterojunctions made of two different compositions often suffer from lattice mismatches, which hinder the efficient transport of charge carriers across interfaces and limit the overall efficiency of the systems.^[^ ^14^, ^15^ ^]^

Notably, the adoption of a metal sulfide homojunction configuration, where identical compositions are maintained on both sides of the interface, offers a compelling alternative. The homojunction ensures perfect lattice matching, resulting in continuous band alignment that effectively eliminates interfacial impedance, thereby featuring a boosted charge transfer and restrained recombination of photogenerated electron‐hole pairs.^[^ ^16^, ^17^ ^]^ For instance, Guo and co‐workers reported a twinned Cd_0.5_Zn_0.5_S nanorod homojunction with type‐II staggered band alignment for solar hydrogen evolution, reaching a remarkable quantum efficiency of 62%.^[^ ^18^ ^]^ The significantly enhanced photoactivity is attributed to the formation of homojunction originating from the alternated zinc‐blende and wurtzite segments to efficiently separate the photogenerated charge carriers. Analogously, homojunctions such as twinned CdS,^[^ ^16^ ^]^ Mn_0.5_Cd_0.5_S,^[^ ^19^ ^]^ amorphous‐crystalline In_2_S_3_,^[^ ^20^ ^]^ amorphous‐crystalline MoS_x_^[^ ^21^ ^]^ have been designed with improved solar‐to‐energy conversion efficiency. It is noteworthy that almost all the fabricated metal sulfide homojunctions feature a type‐II staggered band alignment, which leads to the compromised reduction and oxidation ability of electrons and holes.^[^ ^22^, ^23^ ^]^ Moreover, the accumulated holes in the valence band of metal sulfides are inevitable to corrode catalysts and are deleterious to their photostability under continuous light irradiation.^[^ ^22^, ^24^ ^]^ Alternatively, the recently proposed S‐scheme photocatalyst systems emerge to address the above problems. In these systems, the photoexcited electron‐hole pairs with weak redox ability are recombined to reduce the concentration of holes while the charge carriers with strong redox ability are earned for photocatalytic reaction.^[^ ^23^, ^25^, ^26^, ^27^, ^28^, ^29^ ^]^ Despite the extensive research conducted on sulfide homojunction to enhance photocatalysis, the development of metal sulfide‐based S‐scheme homojunction remains an unexplored frontier.

The semiconductor CdIn_2_S_4_ stands as a promising bimetallic sulfide material due to its superior photostability and optoelectronic properties, as well as its substantial absorption in the visible region, surpassing other metal sulfide semiconductors.^[^ ^30^, ^31^, ^32^, ^33^ ^]^ These critical physicochemical characteristics of CdIn_2_S_4_ have found successful applications in a multitude of advanced photocatalytic processes. On the other hand, aromatic aldehydes are widely used as versatile components for the synthesis of pharmaceuticals and fine chemicals. However, most of them are traditionally produced industrially using stoichiometric oxidants (chromate and permanganate, et al.), a reaction system that still suffers from the drawbacks of high price, high toxicity, and the generation of heavy‐metal wastes, which greatly limits its large‐scale application.^[^ ^30^, ^32^, ^33^ ^]^ Therefore, efficient and sustainable photocatalysts are urging to be explored for the photocatalytic production of aromatic aldehydes from the corresponding alcohols under mild conditions. In this study, we have crafted a CdIn_2_S_4_ S‐scheme homojunction photocatalyst through the straightforward adjustment of polyvinyl pyrrolidone (PVP) quantity during the solvothermal synthesis. This innovative approach entails the encapsulation of 3D CdIn_2_S_4_ octahedra (referred to as o‐CIS) with 2D CdIn_2_S_4_ nanosheets (referred to as n‐CIS). The S‐scheme homojunction within n‐CIS/o‐CIS has been systematically investigated via ultraviolet photoelectron spectroscopy (UPS), femtosecond transient absorption (fs‐TA) spectroscopy, and density functional theory (DFT) characterization, which generates an internal electric field to facilitate the separation of charge carriers. As a result, the n‐CIS/o‐CIS composite exhibits significantly improved photocatalytic activity and stability in the selective oxidation of phenylcarbinol (PhCH_2_OH) to benzaldehyde (PhCHO) when compared to pure n‐CIS and o‐CIS samples. Specifically, the PhCHO production rate of up to 19.9 mmol g^−1^ h^−1^ for n‐CIS/o‐CIS, which is the highest among the reported CdIn_2_S_4_‐based photocatalysts for selective PhCHO production. Our study not only contributes novel insights into the development of metal sulfides featuring S‐scheme homojunction but also underscores their potential to significantly enhance photocatalytic performance and stability under mild conditions, particularly in the realm of selective organic conversions.

## 2 Result and Discussion

**Figure 1:**
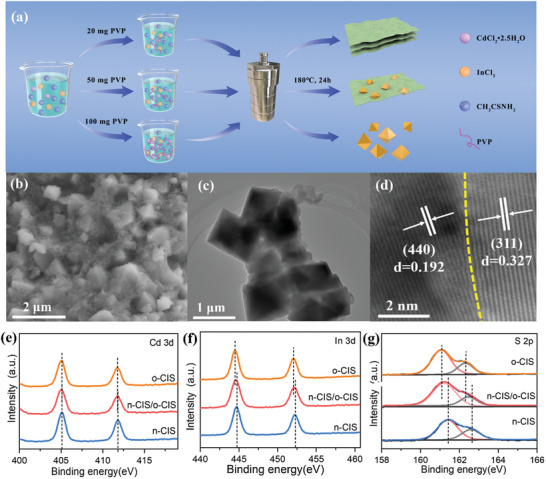
a) Flowchart for the fabrication of n‐CIS, n‐CIS/o‐CIS and o‐CIS. b) SEM, c) TEM, d) HRTEM images of n‐CIS/o‐CIS homojunction. e) Cd 3d, f) In 3d and g) S 2p high‐resolution spectra of n‐CIS, o‐CIS and n‐CIS/o‐CIS homojunction.

The morphologies of CdIn_2_S_4_ materials could be meticulously through a one‐step hydrothermal method. As displayed in **Figure** **1**a, CdIn_2_S_4_ nanosheet, CdIn_2_S_4_ octahedron, and nanosheet/octahedron CdIn_2_S_4_ structure were synthesized facilely by the addition of varying quantities of polyvinyl pyrrolidone (PVP) during the solvothermal process. The morphology and microstructure of these prepared samples were characterized by scanning electron microscopy (SEM) and transmission electron microscopy (TEM). With a relatively low amount of PVP (20 mg), a 2D n‐CIS nanosheet can be obtained, as presented in Figures S1,S2 (Supporting Information). High‐resolution transmission electron microscopy (HRTEM) image (Figure S3, Supporting Information) shows the serried lattice fringes with a d‐spacing of 0.192 nm, corresponding to the (440) crystal plane of the cubic phase CdIn_2_S_4_ (JCPDS No. 27–0060). Energy dispersive X‐ray spectroscopy (EDX) spectrum (Figure S4, Supporting Information) and the associated element mapping images of the n‐CIS (Figure S5, Supporting Information) reveal that the n‐CIS consists of cadmium (Cd), indium (In), and sulfur (S) elements. Increasing the PVP content to 100 mg leads to the formation of the o‐CIS with an octahedral morphology (Figures S6,S7, Supporting Information). The HRTEM image in Figure S8 (Supporting Information) demonstrates a d‐spacing value of 0.327 nm assigned to the (311) crystal plane of the cubic phase CdIn_2_S_4_ (JCPDS No. 27–0060). Three elements (Cd, In and S) of o‐CIS are evenly distributed throughout the octahedrons (Figures S9,S10, Supporting Information). The crystal growth of CIS material is significantly influenced by the PVP dosage. Interestingly, by introducing 50 mg of PVP, a 2D n‐CIS nanosheet‐covered 3D o‐CIS octahedron structure can be achieved, as shown in Figure 1b,c. HRTEM image in Figure 1d and Figure S11 (Supporting Information) reveals distinctive lattice fringes with d‐spacing values of 0.192 and 0.327 nm, attributed to the (440) facet of n‐CIS and the (311) facet of o‐CIS, respectively. This observation confirms the formation of 2D/3D n‐CIS/o‐CIS homojunction with intimate contact. Furthermore, EDX results exhibit homogeneous dispersion of compositions (Cd, In, and S) at the n‐CIS/O‐CIS homojunction (Figures S12,S13, Supporting Information).

The X‐ray diffraction (XRD) patterns in Figure S14 (Supporting Information) indicate that the diffraction peaks of o‐CIS, n‐CIS/o‐CIS, and n‐CIS samples align closely with the distinctive characteristics of the cubic spinel phase of CdIn_2_S_4_ (JCPDS No.27‐0060) and exhibit a high crystallinity. Importantly, no impurity peaks are observed in the XRD patterns of these materials, indicating their exclusive possession of the cubic phase of CIS. To further determine the surface characteristics and elemental valence states of the synthesized samples, X‐ray photoelectron spectroscopy (XPS) was performed. The survey spectrum of n‐CIS/o‐CIS reveals that no peaks of other elements except Cd, In, S, C, O are observed (Figure S15, Supporting Information) and the C and O elements can likely be attributed to the usage of graphite conductive adhesive and the adsorption of atmospheric gaseous species. The high‐resolution core spectra of Cd 3d, In 3d, and S 2p of pure n‐CIS, o‐CIS, and n‐CIS/o‐CIS composite are shown in Figure 1e–g. The peaks observed at 405.1 and 411.6 eV in the Cd 3d spectrum (Figure 1e) are attributed to Cd 3d_5/2_ and Cd 3d_3/2_, respectively.^[^ ^32^ ^]^ The peaks appearing at binding energies of 444.4 and 451.9 eV in the In 3d spectrum (Figure 1f) can be assigned to In 3d_5/2_ and In 3d_3/2_, while the peak observed at 161.2 and 162.4 eV in the S 2p spectrum (Figure 1g) correspond to S 2p_3/2_ and S 2p_1/2_, respectively.^[^ ^32^ ^]^ These results demonstrate that Cd, In, and S are present in their respective valence states of Cd^2+^, In^3+^ and S^2−^ within these samples. Remarkably, it is important to observe that the elemental states of the n‐CIS/o‐CIS homojunction have changed chemically with respect to pure n‐CIS and o‐CIS, suggesting the electronic interactions between n‐CIS and o‐CIS within the homojunction. Specifically, compared with the binding energies of pure n‐CIS, the binding energies of In 3d and S 2p in the n‐CIS/o‐CIS composite display negative shifts. Conversely, in comparison to pure o‐CIS, the binding energies of In 3d and S 2p in the n‐CIS/o‐CIS composite exhibit positive shifts. Moreover, the binding energies of Cd 3d of the n‐CIS/o‐CIS are unchanged in contrast to n‐CIS and o‐CIS. Generally, the binding energy of an element will shift negatively when it obtains electrons and shift positively when it loses electrons. Therefore, the XPS results imply that the charge carriers migrate from o‐CIS to n‐CIS through [In‐S] in the homo‐structure.^[^ ^34^, ^35^ ^]^ Consequently, an interface internal electric field is established within the n‐CIS/o‐CIS composite, directed from o‐CIS to n‐CIS. The interfaceinternal electric field serves as an intrinsic driving force for charge separation and transportation within the n‐CIS/o‐CIS homojunction (for detailed proofs and discussions, refer to subsequent sections).

**Figure 2:**
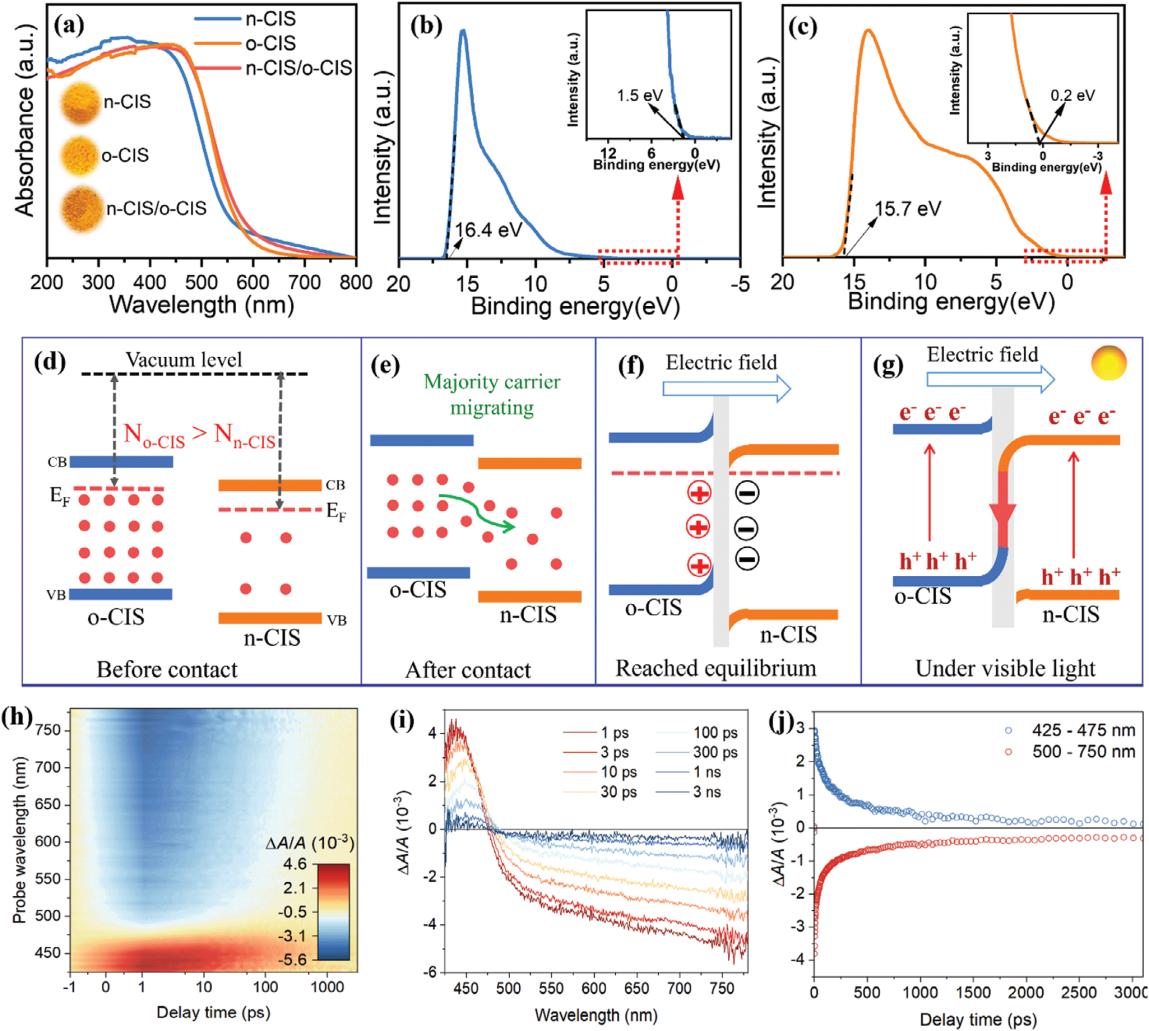
a) UV–vis DRS spectra of n‐CIS, o‐CIS and n‐CIS/o‐CIS homojunction. UPS spectra of b) n‐CIS and c) o‐CIS. d) The Fermi levels of n‐CIS and o‐CIS (the red dots present the majority carriers). e) Internal electric field forming by diffusion of charge carriers from o‐CIS to n‐CIS. f) The band edge bending and internal electric field formed at the interface of the n‐CIS/o‐CIS homojunction. g) The photoexcited charge separation and transportation of the n‐CIS/o‐CIS homojunction under visible light illumination. h–j) Femtosecond transient absorption spectra recorded at indicated delay times measured with 400 nm excitation.

To elucidate the light absorption and band energy structures, a series of experiments were carried out. The optical properties of n‐CIS, n‐CIS/o‐CIS, and o‐CIS photocatalysts were determined by UV–vis diffuse reflectance spectroscopy (UV–vis DRS). All samples exhibited pronounced light absorption within the visible range, which is also consistent with the color of the sample (**Figure** **2**a). Notably, the absorption edge of the o‐CIS catalyst displays a redshift in comparison to n‐CIS, indicating stronger light absorption. The bandgap energies of n‐CIS and o‐CIS photocatalysts were calculated using the formula: αhν = A(hν – E_g_)^1/2^.^[^ ^36^ ^]^ The calculated bandgap energies for n‐CIS and o‐CIS are determined to be 2.37 and 2.25 eV, respectively (Figure S16, Supporting Information). To further explore the band energy positions, Mott‐Schottky experiments were performed. As shown in Figures S17,S18 (Supporting Information), the Mott–Schottky plots for both n‐CIS and o‐CIS photocatalysts exhibit positive slopes, suggesting their n‐type semiconductor nature. The flat band energies of n‐CIS and o‐CIS are −0.30 and −0.46 eV (vs NHE), respectively. Given that the band energy (E_CB_) of n‐type semiconductors is generally negative 0.1 eV than their flat band energy,^[^ ^6^ ^]^ the band energies (E_CB_) of n‐CIS and o‐CIS are found to be −0.40 and −0.56 eV (vs NHE), respectively. Utilizing the formula E_VB_ = E_CB_ + E_g_, the E_VB_ of n‐CIS (1.97 eV) and o‐CIS (1.69 eV) can be obtained, as illustrated in Figure S19 (Supporting Information). Apparently, the n‐CIS/o‐CIS homogeneous junction has an interlaced electronic band structure. Therefore, both the band‐band (type‐II) transfer and the S‐scheme transfer mechanisms for the photoexcited charge separation and transportation mechanism within the n‐CIS/o‐CIS are possible (Figure S20, Supporting Information). These transfer mechanisms both facilitate efficient photoexcited charge separation. However, further experimental investigations are required to precisely elucidate the exact charge transfer mechanism operative in the n‐CIS/o‐CIS photocatalyst.

However, in the context of the type‐II transfer mechanism, the reducing potential of transferred electrons residing in the CB of n‐CIS and the oxidizing capacity of transferred holes within the VB of o‐CIS are compromised. Conversely, within the S‐scheme transfer mechanism, the high redox potentials of electrons in the CB of o‐CIS and holes in the VB of n‐CIS remain preserved. Compared with the •O_2_^−^ generation potential (O_2_/•O_2_^−^ = −0.33 eV), the electrons in the CB of o‐CIS possess a higher propensity for reducing O_2_ to form •O_2_^−^ when compared to their counterparts in n‐CIS. Consequently, the type‐II band‐band transfer mechanism appears less conducive to the generation of •O_2_^−^, while the S‐scheme transfer mechanism emerges as a more favorable route for •O_2_^−^ production. Moreover, within the S‐scheme mechanism, the photogenerated holes reserving in the VB of n‐CIS, characterized by their heightened oxidation potential relative to o‐CIS, can be deemed advantageous for the oxidation of compounds such as phenylcarbinol.

As it is well‐established, the charge transfer between two semiconductors is determined by the Fermi level. Generally, charge carriers diffuse from a semiconductor with a higher Fermi level to one with a lower Fermi level until an equilibrium is reached, thereby establishing an internal electric field at the semiconductor interface.^[^ ^34^, ^37^ ^]^ This IEF serves as a pivotal factor governing the process of photoexcited charge separation and transfer within junction photocatalysts possessing interlaced electronic band structures. Thus, ultraviolet photoelectron spectroscopy (UPS) was performed to measure the Fermi levels of n‐CIS and o‐CIS semiconductors based on the equation: Ф = E_vac_ – E_F_, where Ф represents the work function, E_vac_ stands for the vacuum level, and E_F_ denotes the Fermi level.^[^ ^5^, ^6^ ^]^ The results are displayed in Figure 2b,c. The work functions of n‐CIS and o‐CIS are ≈6.3 and 5.7 eV, respectively. Correspondingly, the Fermi levels of n‐CIS and o‐CIS are ≈−6.3 and −5.7 eV (vs vacuum level), respectively.

Moreover, the carrier densities of both n‐CIS and o‐CIS are calculated based on the Mott–Schottky plots in Figures S17,S18 (Supporting Information) according to the formula: N_D_ = (2/e_0_εε_0_)[dU_FL_/d(1/C^2^)] (ε is the dielectric constant (Figure S21, Supporting Information), ε_0_ = 8.85 × 10^−12^ F cm^−2^, C is the capacitance, e = 1.6 × 10^−19^ C, and U_FL_ is the potential as 1/C^2^ → 0 based on the Mott–Schottky curve).^[^ ^5^, ^6^, ^38^ ^]^ Notably, the o‐CIS sample always shows higher carrier density (N_D_) than the n‐CIS across various frequencies (Figure S22, Supporting Information). Specifically, the carrier densities of the o‐CIS are ≈1.4 × 10^21^, 3.4 × 10^21^, and 7.6 × 10^21^ cm^−3^ at 600, 800, and 1000 Hz, respectively. In contrast, the carrier densities of the n‐CIS are ≈3.1 × 10^20^, 7.4 × 10^20^, and 1.7 × 10^21^ cm^−3^, respectively. Overall, the carrier density of o‐CIS (N_o‐CIS_) is ≈4.5 times greater than that of n‐CIS (N_n‐CIS_). The o‐CIS not only possesses a higher Fermi level but also contains a larger carrier density when compared to n‐CIS (Figure 2d). Therefore, the majority of carriers (electrons for *n*‐type semiconductors) in the o‐CIS undergo rapid diffusion toward n‐CIS as waterfalls until equilibrium is attained (Figure 2e,f). Throughout this migration process, the surface of n‐CIS becomes negatively charged due to electron accumulation, leading to a downward bending of the band edge. Conversely, the surface of o‐CIS becomes positively charged owing to electron loss, causing the band edge to bend upward. Upon achieving equilibrium, an IEF with the direction of o‐CIS → n‐CIS is established at the n‐CIS/o‐CIS interfaces (Figure 2f).

Based on the above result, the detailed charge transfer mechanism in n‐CIS/o‐CIS homojunction can be revealed. Specifically, under visible light irradiation, the established IEF and resultant band bending serve dual roles. They not only promote the recombination of the photoexcited electrons in the CB of n‐CIS and the photoexcited holes in the VB of o‐CIS at the interfaces of n‐CIS/o‐CIS composite but also act as a hindrance to the recombination of the photoexcited electrons in the CB of o‐CIS and the photoexcited holes in the VB of n‐CIS. This dual effect prevents the type‐II transfer mechanism while promoting the S‐scheme transfer (Figure 2g). To illustrate this point, consider the behavior of photoexcited electrons on o‐CIS. These electrons have the propensity to remain within o‐CIS rather than transferring to n‐CIS due to several key factors: 1) The elevated energy requirement for electron transition from the CB of o‐CIS to that of n‐CIS, attributed to the upward position of the CB edge on o‐CIS. 2) Coulomb repulsion between the photoexcited electrons residing on o‐CIS and the internal electric field further discourages their migration to n‐CIS. 3) Additionally, Coulombic repulsion exists between the photoexcited electrons situated on o‐CIS and those within n‐CIS. Similarly, photoexcited holes within n‐CIS exhibit a similar behavior, tending to remain within n‐CIS. Consequently, the type‐II transfer mechanism is hindered within the n‐CIS/o‐CIS homojunction photocatalyst.

On the contrary, within the n‐CIS/o‐CIS homojunction photocatalysts, the photoexcited electrons in n‐CIS can effectively engage in recombination with the photoexcited holes present in o‐CIS due to the bending energy band and Coulomb attraction. The synergetic interaction of band bending, IEF, and Coulomb force simultaneously provides the driving force for the S‐scheme transfer mechanism within the n‐CIS/o‐CIS system. Thus, the photoexcited electrons with a lower reduction potential in n‐CIS and the holes with a weak oxidation potential in o‐CIS are recombined. Simultaneously, the photogenerated electrons with a higher reduction potential in o‐CIS and the holes with a stronger oxidation potential on the n‐CIS are reserved and spatially separated for photocatalytic reactions.

To further investigate the dynamics and mechanism of charge transfer, we conducted femtosecond transient absorption (fs‐TA) spectroscopy for the n‐CIS/o‐CIS system. Figure 2h illustrates the pseudo‐color TA plot of n‐CIS/o‐CIS. Upon excitation by a pump pulse at 400 nm, the fs‐TA spectrum of n‐CIS/o‐CIS reveals a distinctive peak at ≈440 nm, exhibiting varying intensities at different time delays, which is attributed to excited‐state absorption (ESA) (Figure 2h).^[^ ^39^, ^40^ ^]^ Simultaneously, a broad ground‐state bleach (GSB) band spanning 475–800 nm is evident (Figure 2i). The ESA exhibits a positive signal, which is then quickly replaced by a strong bleaching (Figure 2i,j). The extended bleaching signals observed at longer wavelengths can be attributed to sub‐bandgap trap‐state absorption and indirect bandgap transitions, aligning with the characteristics of steady‐state absorption spectra.^[^ ^41^ ^]^ Subsequent elucidation of the decay kinetics of photogenerated carriers involved fitting the temporal distribution of the detected transient absorption, revealing a multi‐exponential decay pattern (Figure 2j; Figure S23, Supporting Information). The results of fitting the decay kinetic curves at 440 nm unveil three distinct lifetimes, spanning from a few picoseconds to several nanoseconds (Figure S23, Supporting Information).^[^ ^39^, ^42^ ^]^ This kinetic analysis indirectly verifies the accelerated charge separation in the constructed S‐scheme heterojunction once again (Figure S24, Supporting Information).^[^ ^43^ ^]^

**Figure 3:**
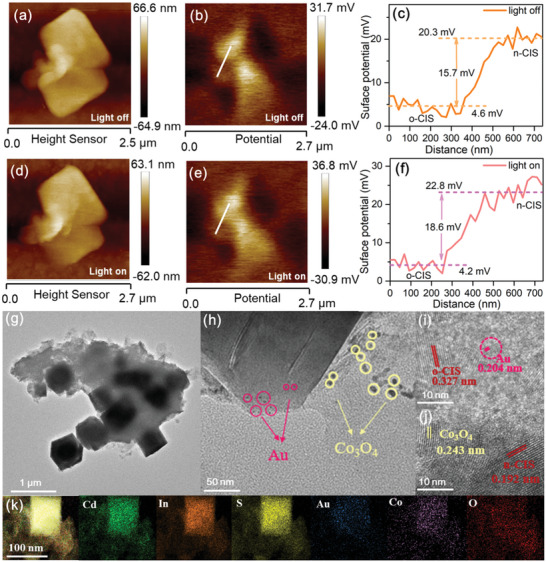
KPFM measurement of n‐CIS/o‐CIS homojunction. a,d) Height images, b,e) potential images and e,f) surface potential curves of n‐CIS/o‐CIS homojunction surface in the dark and under illumination. g) TEM, h) magnified TEM, i,j) HRTEM, and k) EDX element mapping images of the Co_3_O_4_/n‐CIS/o‐CIS/Au sample.

To further confirm the efficient charge separation and transfer process in the n‐CIS/o‐CIS homojunction, Kelvin probe force microscopy (KPFM) was applied to study the space charge distribution of the n‐CIS/o‐CIS catalyst in the dark and under light irradiation.^[^ ^29^ ^]^ The height image (**Figure** **3**a) shows that the selected region is the interface between o‐CIS and n‐CIS in the homojunction. As shown in Figure 3b,c, the distribution of the surface potentials of the two components in the dark indicates that n‐CIS has a larger surface potential in the homojunction than o‐CIS, creating a gap of 15.7 mV between them. According to the formula: *V*_CPD_ = −(Φ_tip_ − Φ_sample_)∕e (*V*_CPD_ represents the contact potential difference between the tip and the sample surface; Φ_tip_ and Φ_sample_ represent the work functions of the tip and sample), the work function of n‐CIS at the homogeneous junction is larger than that of o‐CIS, which is also consistent with the above UPS results and the theoretical calculations. Under light illumination, the surface potentials at the n‐CIS/o‐CIS junctions in the same region changed significantly (Figure 3d–f). A decrease of the surface potential from 4.6 to 4.2 mV was observed at o‐CIS, while a significant increase from 20.3 to 22.8 mV was detected at n‐CIS, indicating the generation and subsequent separation of photogenerated electron‐hole pairs. The decreased surface potential of o‐CIS indicates electron enrichment, whereas the increased surface potential of n‐CIS indicates hole accumulation. The change in KPFM surface potential caused by light irradiation at the n‐CIS/o‐CIS homojunction further confirms the feasibility of the S‐scheme charge transfer mechanism.

To visually observe the S‐scheme mechanism, the direction of electron transfer of the n‐CIS/o‐CIS was investigated by the simultaneous photo‐deposition of Co_3_O_4_ and Au on the surface of the n‐CIS/o‐CIS homojunction. The Co_3_O_4_/n‐CIS/o‐CIS/Au sample shows almost the same morphology as the n‐CIS/o‐CIS (Figure 3g), indicating the stability of the n‐CIS/o‐CIS homojunction during the photo‐deposition process. After magnification, as shown in Figure 3h, Au nanoparticles and Co_3_O_4_ fragments can be detected on the surfaces of the o‐CIS and the n‐CIS, respectively. HRTEM images show that the lattice spacing of 0.243 nm observed near the n‐CIS nanosheet belongs to the (311) crystal plane of Co_3_O_4_ (JCPDS No. 43–1003, Figure 3i), and the lattice spacing of 0.204 nm in the outer layer of the o‐CIS octahedron can be attributed to the (200) crystal plane of Au (JCPDS No. 04–0784, Figure 3j). The reduced cocatalyst Au and the oxidized cocatalyst Co_3_O_4_ are selectively deposited on n‐CIS nanosheets and octahedra o‐CIS, respectively. Moreover, the EDX mapping results in Figure 3k show that Au has a relatively small distribution range mainly on octahedral o‐CIS, while Co and O have a relatively large distribution range on n‐CIS nanosheets. The in situ photo‐deposition characterization visually proves the feasibility of the S‐scheme transfer mechanism of the n‐CIS/o‐CIS homojunction.

**Figure 4:**
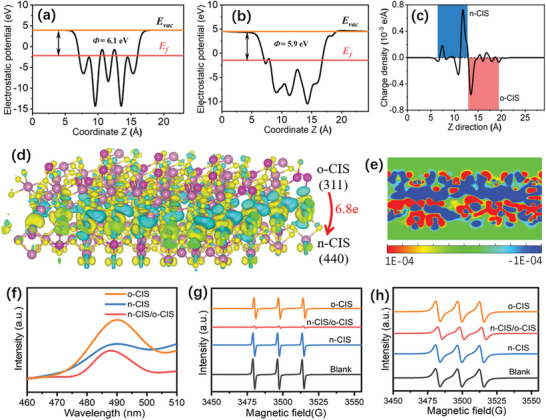
Electrostatic potentials of a) n‐CIS and b) o‐CIS surfaces. c) The planar‐averaged electron density difference over the n‐CIS/o‐CIS heterojunction. Simulated differential charge density distribution at the interface between n‐CIS and o‐CIS with an isosurface of 1.5 × 10^−3^ e Å^−3^. d) side view. (The charge accumulation is shown as the yellow region, and the charge depletion is shown as the cyan region.) e) 2D map of differential charge density map at the interface between n‐CIS and o‐CIS. f) PL spectra of n‐CIS, o‐CIS and n‐CIS/o‐CIS homojunction. EPR spectra of g) TEMPO‐e^−^ and h) TEMPO‐h^+^ of the n‐CIS, o‐CIS and n‐CIS/o‐CIS samples under visible light irradiation (blank means TEMPO in the reaction system without catalysts under visible light irradiation).

Density Functional Theory (DFT) calculations were carried out to provide a comprehensive exploration of the charge carrier transfer mechanisms inherent to the n‐CIS/o‐CIS homojunction. Electrostatic potentials of n‐CIS and o‐CIS surfaces were first evaluated which affords us a window into atomic‐level charge transfer phenomena. Here, we introduce the Ф value defined as the difference between E_vac_ and E_F_, wherein a larger Ф corresponds to a lower E_F_. As shown in **Figure** **4**a,b, the n‐CIS exhibits a relatively greater Ф value (6.1 eV) compared to o‐CIS (5.9 eV), which signifies that the E_F_ of the n‐CIS is lower than that of o‐CIS. Therefore, the majority of carriers migrate from o‐CIS into n‐CIS, ultimately culminating in the establishment of an internal electric field, oriented from o‐CIS toward n‐CIS, at the interface of the n‐CIS/o‐CIS homojunction.^[^ ^5^, ^6^ ^]^ The planar‐averaged charge density difference along the Z direction between n‐CIS and o‐CIS is displayed to further analyze the charge transfer on the interface of the n‐CIS/o‐CIS homojunction (Figure 4c). The change of position‐dependent charge density difference at interface evidence that the o‐CIS donates electrons to n‐CIS. It can be confirmed by the simulated differential charge density distribution at the interface between n‐CIS and o‐CIS (Figure 4d,e; Figure S25, Supporting Information). Approximately 6.8 electrons are observed to migrate from o‐CIS (311) into n‐CIS (440), a result that finds concordance with the above experimental observations.

To provide a more comprehensive understanding of the dynamics governing photoexcited charge separation and recombination across the n‐CIS/o‐CIS S‐scheme homojunction, the photoelectrochemical (PEC) method, steady‐state photoluminescence (PL), time‐resolved PL (TRPL) and electron paramagnetic resonance (EPR) techniques were performed.^[^ ^44^, ^45^ ^]^ The photocurrent intensity of the n‐CIS/o‐CIS is significantly higher than that of pure n‐CIS and o‐CIS (Figure S26, Supporting Information). Furthermore, the electrochemical impedance spectrum (EIS) reveals the n‐CIS/o‐CIS composite to possess the smallest arc radius (Figure S27, Supporting Information). Additionally, the linear sweep voltammetry (LSV) curves, obtained under visible light irradiation, show an enhanced cathodic current density for the n‐CIS/o‐CIS photocatalyst within the potential region of −1.0 – 0 V (V vs RHE), when contrasted with n‐CIS and o‐CIS sample (Figure S28a, Supporting Information). Moreover, the corresponding Tafel slope of n‐CIS/o‐CIS (62 mV dec^−1^) is smaller than that of n‐CIS (99 mV dec^−1^) and o‐CIS and (108 mV dec^−1^) (Figure S28b, Supporting Information), suggesting that the n‐CIS/o‐CIS homojunction is more efficient in accelerating the kinetics of the reaction compared to the pure n‐CIS and o‐CIS. Collectively, these results indicate that the significant enhancement of photogenerated charge carrier separation is achieved through the formation of the n‐CIS/o‐CIS S‐scheme homojunction, which is beneficial to improving the photocatalytic activity of the system.

Meanwhile, photoluminescence (PL) provides valuable insights into the recombination dynamics of photoexcited electron‐hole pairs.^[^ ^46^, ^47^, ^48^ ^]^ The PL intensity of the n‐CIS/o‐CIS composite is lower than that of both n‐CIS and o‐CIS (Figure 4f), indicating the inhibited recombination charge carries. Subsequently, time‐resolved PL (TR‐PL) decay spectra were further performed to study the photogenerated charge carriers transfer dynamics (Figure S29, Supporting Information). The decay plots are fitted via a biexponential kinetics function (I(t) = A_1_exp(‐t/τ_1_) + A_2_exp(‐t/τ_2_)), in which A_1_ and A_2_ are the corresponding amplitudes, τ_1_ is nonradiative recombination from charge carriers in vacancies or other defects, and τ_2_ is recombination from free excitons within the samples.^[^ ^49^ ^]^ The average lifetime (τ_a_) value is computed by the following formula: τ_a_ = (A_1_τ_1_^2^ + A_2_τ_2_^2^)/(A_1_τ_1_ + A_2_τ_2_).^[^ ^49^ ^]^ The calculated average lifetime for n‐CIS, o‐CIS, and n‐CIS/o‐CIS samples are 0.869, 1.129, and 1.135 ns, respectively (Table S1, Supporting Information). The average lifetime of n‐CIS/o‐CIS exceeds that of both n‐CIS and o‐CIS. However, it is noteworthy that the n‐CIS/o‐CIS exhibits a shorter τ_1_ value (1.012 ns) than n‐CIS (4.253 ns) and o‐CIS (5.504 ns), while simultaneously presenting a longer τ_2_ value (4.786 ns) than n‐CIS (0.830 ns) and o‐CIS (0.994 ns). These results indicate that the electron‐hole recombination of the n‐CIS/o‐CIS is restrained,^[^ ^34^ ^]^ and the charge carriers are trapped by the defects (The existence of defects in the n‐CIS/o‐CIS is demonstrated by solid electron paramagnetic resonance (Figure S30, Supporting Information).

To further reveal the charge separation efficiency veritably, the in situ electron paramagnetic resonance (in situ EPR) technique for detecting holes (h^+^) and electrons (e^−^) was employed using 2,2,6,6‐Tetramethylpiperidinooxy (TEMPO) as a charge trapping agent.^[^ ^34^ ^]^ This technique allows for the detection of both holes (h^+^) and electrons (e^−^) within the system, as the EPR signal intensity diminishes when TEMPO interacts with either electrons or holes. Namely, a weaker EPR signal indicates a higher quantity of charge carriers successfully separated and reaching the surface of the photocatalysts. As depicted in Figure 4g, in comparison to a blank reference (TEMPO in the reaction system without catalysts under visible light irradiation), the EPR signal intensity for trapping electrons is reduced in the presence of different samples, with the n‐CIS/o‐CIS catalyst exhibiting the least intensity amount these samples. Analogous trends also can be observed in the case of detecting holes (Figure 4h). These results demonstrate that the n‐CIS/o‐CIS S‐scheme homojunction can significantly promote the separation and transportation of the photoexcited charge carriers to the surface of the catalyst, ultimately facilitating photocatalytic redox reactions.

**Figure 5:**
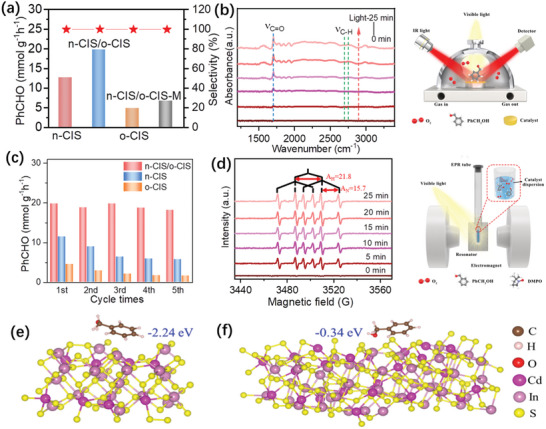
a) Photocatalytic selective oxidation of phenylcarbinol for PhCHO production and PhCHO selectivity over different samples under visible light irradiation. c) The reusability of the n‐CIS/o‐CIS homojunction for photocatalytic phenylcarbinol oxidation. b) Schematic diagrams of in situ diffuse reflection infrared Fourier transform spectroscopy (in situ DRIFTS) and corresponding spectra on n‐CIS/o‐CIS for photocatalytic selective of PhCH_2_OH under visible light irradiation. d) Schematic diagrams of in situ EPR and EPR spectra on photocatalysts under visible light irradiation. e) n‐CIS and f) o‐CIS surfaces adsorbed with benzyl alcohol.

The photocatalytic performance of these samples was evaluated by selective oxidation of phenylcarbinol (PhCH_2_OH) to benzaldehyde (PhCHO) under visible light irradiation. **Figure** **5**a shows PhCHO production and selectivity over these photocatalysts under different reaction conditions. The structure of the benzaldehyde product is demonstrated by ^1^H NMR (nuclear magnetic resonance) spectrum (Figure S31, Supporting Information). Control experiments indicate that no PhCHO product is detected in the absence of photocatalysts or under conditions of darkness. The n‐CIS/o‐CIS sample exhibits superior reactivity in the selective oxidation of phenylcarbinol in comparison to both n‐CIS and o‐CIS. Specifically, the n‐CIS/o‐CIS composite achieves a remarkable PhCHO production rate of up to 19.9 mmol g^−1^ h^−1^, representing a substantial enhancement of 1.6‐fold and 4.1‐fold relative to the rates attained by n‐CIS and o‐CIS, respectively. Turnover number (TON) of the n‐CIS/o‐CIS after 2 h of photocatalytic reaction was calculated to be ≈19, which is ≈1.6 and 3.8 times of n‐CIS (12) and o‐CIS (5), respectively. In addition, the selectivity of the reaction reaches an exceptional level of 99.9%. Moreover, the n‐CIS/o‐CIS sample outperforms the mixture of n‐CIS and o‐CIS (n‐CIS/o‐CIS‐M), which is prepared by the grinding method. This result suggests the pivotal role played by the formation of the n‐CIS/o‐CIS homojunction characterized by close contact, as opposed to the mere physical mixture of components. Notably, the photocatalytic activity of the n‐CIS/o‐CIS sample is the highest among the reported CdIn_2_S_4_‐based photocatalysts for selective PhCHO production (Table S2, Supporting Information). Subsequently, the photocatalytic performance of n‐CIS/o‐CIS homojunction at different wavelengths was investigated (Figure S32, Supporting Information). Clearly, the photocatalytic activities of the n‐CIS/o‐CIS at different wavelengths align seamlessly with its optical absorption spectrum, suggesting the photocatalytic process for selective oxidation of phenylcarbinol. The apparent quantum efficiency (AQE) for PhCHO production over the n‐CIS/o‐CIS at a wavelength of 450 nm is ≈2.3%.

Furthermore, it is well‐established that metal sulfides are often susceptible to photo‐corrosion under light irradiation due to the presence of photogenerated holes. Therefore, we carried out recycling experiments to assess their photostability, while simultaneously scrutinizing the crystal phase and morphology of the samples before and after the photocatalytic reaction. After five cycles of use, both n‐CIS and o‐CIS samples exhibit an obvious photoactivity decrease due to the photo‐corrosion. A high Cd^2+^ concentration, 6.3 mg L^−1^ for n‐CIS and 1.5 mg L^−1^ for o‐CIS, in the solution after the reaction is detected by inductively coupled plasma optical emission spectroscopy (ICP‐OES). Remarkably, no significant loss of activity is observed for the n‐CIS/o‐CIS photocatalyst (Figure 5c) and the Cd^2+^ concentration in the reaction liquid is measured to be ≈0.1 mg L^−1^. The slight reduction in activity observed may be attributed to the loss of photocatalyst material during the recycling tests. At the same time, after the photocatalytic reaction, the used n‐CIS/o‐CIS sample exhibits a similar XRD pattern (Figure S33, Supporting Information) and morphology (Figure S34, Supporting Information) with the fresh n‐CIS/o‐CIS. This indicates that the crystal phase and micro‐structure of the n‐CIS/o‐CIS sample remain unchanged after the photocatalytic reaction, suggesting that the n‐CIS/o‐CIS is a relatively stable catalyst during the reaction process. Moreover, the n‐CIS/o‐CIS also exhibits the universality for selective oxidation of multiple aromatic alcohols with different substituents into the corresponding aromatic aldehydes, as shown in Table S3 (Supporting Information).

Both superoxide radicals (•O_2_^−^) and photogenerated holes (h^+^) have been demonstrated as the primary reactive species governing the selective oxidation of alcohols. To determine the active species responsible for the phenylcarbinol oxidation over the n‐CIS/o‐CIS, control experiments were performed by adding p‐benzoquinone (BQ) and triethanolamine (TEOA) as scavengers to react with •O_2_^−^ and h^+^, respectively. As shown in Figure S35 (Supporting Information), the PhCHO production is significantly reduced when either BQ, argon (Ar), or TEOA is introduced, corroborating the involvement of both •O_2_^−^ and h^+^ in the conversion of phenylcarbinol to benzaldehyde. This is consistent with the EPR results above, in which h^+^ is detected (Figure 4h). Furthermore, the •O_2_^−^ radical is also detected over n‐CIS, o‐CIS and n‐CIS/o‐CIS under visible light by EPR spectra (Figure S36, Supporting Information). The results show that more superoxide radicals can be produced on the n‐CIS/o‐CIS than n‐CIS and o‐CIS, manifesting excellent charge separation ability of the n‐CIS/o‐CIS S‐scheme homojunction. Furthermore, the S‐scheme transfer mechanism operative within the n‐CIS/o‐CIS sample facilitates the retention of photoexcited electrons possessing high reduction potential, thus favoring the formation of •O_2_^−^. This is consistent with the above results of photocatalytic activity.

In situ diffuse reflection infrared Fourier transform spectroscopy (in situ DRIFTS) was employed to further study the interactions between reactants and n‐CIS/o‐CIS sample under visible light irradiation (Figure 5b). With the extension of the light irradiation time, an obvious peak at 1710 cm^−1^ (ν_C═O_) corresponding to the C═O of PhCHO appeared.^[^ ^50^, ^51^ ^]^ Notably, the intensity of this peak exhibits a pronounced increase as the illumination time is prolonged. Additionally, a band at 1615 cm^−1^, attributed to the phenyl ring vibration, can also be detected remarkedly. The two peaks located at 2700–2800 cm^−1^ are assigned to the ν_C─H_ of the aldehyde group.^[^ ^51^ ^]^ These results demonstrate that PhCH_2_OH molecules are converted into PhCHO over the n‐CIS/o‐CIS sample under visible light irradiation.

To gain further insights into the intermediate radicals generated during the photocatalytic selective oxidation of phenylcarbinol, in situ EPR technique was performed using DMPO (5,5‐dimethyl‐1‐pyrroline N‐oxide) as a typical molecular probe (Figure 5d). When the reaction systems containing n‐CIS, n‐CIS/o‐CIS, and o‐CIS photocatalysts are irradiated under visible light, six EPR signals of the characteristic peaks of the free radicals at the carbon center can be observed. The results show *A*_H_ = 21.8 G and *A*_N_ = 15.7 G, corresponding to the produced DMPO‐•CH(OH)Ph intermediate during the photocatalytic selective oxidation of phenylcarbinol.^[^ ^49^, ^52^ ^]^ This suggests that the phenylcarbinol can be converted into aldehyde via the radical‐induced reaction mechanism. Remarkably, a previous study reported by Zhao et al. claimed that the oxidation of phenylcarbinol may also follow an oxygen atom transfer pathway,^[^ ^53^ ^]^ in which the C─O bond of the alcohol is cleaved, with dioxygen facilitating the formation of a new C═O bond in the aldehyde product while simultaneously generating H_2_O_2_. To verify the presence of this additional reaction pathway, we probed the production of H_2_O_2_ after the photocatalytic oxidation reaction. The results in Figure S37 (Supporting Information) show that H_2_O_2_ can be detected in the reaction system, providing strong evidence for the coexistence of both mechanisms for aldehyde production from phenylcarbinol under light irradiation.

The adsorption of PhCH_2_OH reactants on the surface of the catalyst is of crucial importance for the photocatalytic oxidation reaction. Therefore, we performed DFT calculations to study the PhCH_2_OH adsorption ability of n‐CIS and o‐CIS catalysts. The results displayed in Figure 5e,f reveal that these two samples exhibit significantly different adsorption abilities toward PhCH_2_OH. Specifically, the adsorption energy of PhCH_2_OH over n‐CIS is ca. 8 times greater than that observed for the o‐CIS sample. In the case of the n‐CIS/o‐CIS composite, the strong adsorption ability toward reactants over n‐CIS is also favorable for promoting the oxidation of PhCH_2_OH. This is attributed to the S‐scheme charge transfer mechanism in the n‐CIS/o‐CIS composite, which will retain photogenerated holes with strong oxidation ability on the n‐CIS side. Therefore, combining the above results and analysis, the adsorbed PhCH_2_OH reactants on the surface of n‐CIS could be directly converted into products by holes (Path‐A in Figure S38, Supporting Information). On the other hand, the o‐CIS in n‐CIS/o‐CIS composite with low adsorption ability of reactants will leave sufficient active sites for reducing oxygen molecules into superoxide radicals, thus enhancing the oxidation of PhCH_2_OH (Path‐B in Figure S38, Supporting Information) and promoting overall photocatalytic performance.

## 3 Conclusion

In summary, n‐CIS/o‐CIS S‐scheme homojunction photocatalysts were prepared by a facile one‐step solvothermal method. EPR, UPS, KPFM, and DFT analyses consistently support the assertion that the charge transfer mechanism operative in the n‐CIS/o‐CIS system follows an S‐scheme pathway, rather than a band–band transfer mechanism. As a result, the n‐CIS/o‐CIS homojunction demonstrates markedly improved photocatalytic activity and stability in comparison to their counterparts, n‐CIS and o‐CIS, in the selective oxidation of phenylcarbinol into PhCHO under visible light irradiation. Both h^+^ and •O_2_^−^ species play pivotal roles in driving the conversion of phenylcarbinol and two different reaction mechanisms for aldehyde production from phenylcarbinol are confirmed. Our findings emphasize the critical importance of controlling the separation and migration of photogenerated carriers for facilitating the efficacy of photocatalysts in redox‐driven reaction systems powered by photocatalysis.

## 4 Experimental Section

### Materials

Cadmium chloride hemi (pentahydrate) (CdCl_2_·2.5H_2_O, ≥99.0%), Indium chloride (InCl_3_, >99.99%), thioacetamide (C_2_H_5_NS, ≥99.0%), polyvinyl pyrrolidone ((C_6_H_9_NO)n), ethanol (C_2_H_5_OH, water ≤0.03%), methanol (CH_3_OH, 99.5%), benzyl alcohol (C_7_H_8_O, ≥99%), sodium sulfate (Na_2_SO_4_, ≥ 98%), 5,5‐Dimethyl‐1‐pyrroline N‐oxide (DMPO, ≥98%), acetonitrile (CH_3_CN, >99%), 2,2,6,6‐Tetramethylpiperidinooxy (TEMPO, 99%) and other reagents were directly used in the experiments without any further purification. Deionized water used in the synthesis was from local sources.

### Preparation of CdIn_2_S_4_ Nanosheets, Octahedron, and Nanosheets/Octahedron Homojunction

Dispersed CdIn_2_S_4_ nanosheets and octahedron were synthesized by a solvothermal method. In a typical procedure, 45.672 mg CdCl_2_·2.5H_2_O, 88.5 mg InCl_3_, 120 mg C_2_H_5_NS, and polyvinyl pyrrolidone (PVP) were successively dissolved in 16 mL deionized water and 16 mL ethanol under continuous stirring. After stirring for ≈60 min, the mixture was transferred into a Teflon‐lined autoclave (Anhui Kemi Instrument Co., Ltd.) and kept at 180 °C for 24 h. The obtained yellow precipitates were naturally cooled at room temperature and collected by centrifugation. Finally, the collected product was washed deionized water and ethanol alternately and dried in a vacuum oven at 60 °C for 10 h. CdIn_2_S_4_ nanosheets (n‐CIS), octahedron (o‐CIS), and nanosheets/octahedron homojunction (n‐CIS/o‐CIS) could be easily synthesized by changing the amount of PVP with 20, 100, and 50 mg.

### Characterization

The crystal phases of the samples were analyzed by X‐ray diffraction (XRD) on a Bruker D8 Advance X‐Ray Diffractometer with Ni filtered Cu Kα radiation (*λ* = 1.5406 Å) at a voltage of 40 kV and a current of 40 mA. The optical properties of the sample were characterized by UV–vis diffuse reflectance spectroscopy (DRS, Shimadzu UV‐3600), in which BaSO_4_ was used as the internal reflectance standard. Electron paramagnetic resonance (EPR) spectra were measured on the Steady High Magnetic Field Facilities, High Magnetic Field Laboratory, CAS, and the same light source as that for the photocatalytic reaction. X‐ray photoelectron spectroscopy (XPS) and ultraviolet photoelectron spectroscopy (UPS) tests were conducted on a Thermo Scientifific ESCA Lab250 spectrometer. Scanning electron microscopy (SEM) and energy‐dispersive X‐ray spectroscopy (EDX) were applied to determine the microstructure and elemental composition of the sample on an FEI Nova NANOSEM 450 spectrophotometer. The microstructures of the photocatalysts were measured by transmission electron microscopy (TEM, JEM 2100) and high‐resolution transmission electron microscopy (HRTEM). Photoluminescence (PL, F97 Pro) emission spectra were recorded using a fluorescence spectrometer and time‐resolved photoluminescence spectroscopy (TR‐PL) was analyzed on an Edinburgh FS5 fluorescence spectrophotometer. The femtosecond transient absorption (fs‐TA) is based on a regenerative amplified Ti:sapphire laser system from Coherent (800 nm and 1000 Hz repetition rate), nonlinear frequency mixing techniques, and the Femto‐TA100 spectrometer (Time‐Tech Spectra). The dielectric constant was measured by the impedance analyzer (Agilent 4294A). Atomic force microscope (AFM) images and Kelvin probe force microscopy (KPFM) images were captured by Bruker MultiMode 8. The Cd content was detected by an inductively coupled plasma optical emission spectroscopy (ICP‐OES, PerkinElmer ICP 2100). ^1^H nuclear magnetic resonance (NMR) spectra were obtained on Bruker ASCEND 600.

### Structure and DFT Computational Detail

Density function theory (DFT) calculations were performed by using the CP2K‐9.1 package. Perdew–Burke–Ernzerh (PBE) of functional was used to describe the system. Unrestricted Kohn–Sham DFT had been used as the electronic structure method in the framework of the Gaussian and plane waves (GPW) way. The Goedecker–Teter–Hutter (GTH) pseudopotentials and Double‐ζ molecularly optimized basis sets (DZVP‐MOLOPT‐GTH) had been used for all elements. A plane‐wave energy cutoff of 400 Ry had been employed. The geometries were optimized using the Broyden‐Fletcher‐Goldfarb‐Shanno (BFGS) algorithm, and the convergence criterion for the forces was set to 4.5 × 10^−4^ bohr/hartree. A vacuum layer of 15 Å was constructed to eliminate interactions between periodic structures of surface models.

The adsorption energy (ΔE_ads_) of benzyl alcohol adsorption on surface is defined as ΔE_ads_ = E(*benzyl alcohol) – E(*) – E(benzyl alcohol), where E(*benzyl alcohol) and E(*) are the total energy of surface systems with and without benzyl alcohol, respectively, E(benzyl alcohol) is the energy of an isolated benzyl alcohol molecule. According to this definition, negative adsorption energy suggests that the adsorption process is exothermic and the adsorption system is thermodynamically stable. Contrarily, a positive value corresponds to an endothermic and unstable adsorption.

### Photoelectrochemical Test

The photoelectrochemical tests were carried out on a three‐electrode system (CHI‐660E). The photocurrent, electrochemical impedance spectroscopy, open circuit potential, and Mott–Schottky experiments were carried out using a conventional three‐electrode cell (an as‐prepared CdIn_2_S_4_ electrode (50 mm × 50 mm) as the working electrode, a Pt wire as the counter electrode, and an Ag/AgCl electrode as the reference electrode). The electrolyte for photocurrent, open circuit potential, EIS, and M‐S experiments was 0.1 m Na_2_SO_4_ solution. The light source was the same as that for the photocatalytic reaction.

### In Situ DRIFT Test

In situ diffuse reflectance infrared Fourier transform spectroscopy (DRIFT) measurements were performed on a Nicolet 8700 FTIR spectrometer using a KBr window. The dried sample and a trace amount of benzyl alcohol solution were first placed into the in situ reactor, and then the reactor was mounted behind the sample chamber, and oxygen was introduced into the chamber. After adsorption of benzyl alcohol and oxygen for 30 min, the reactor was illuminated with a 300 W Xenon lamp (λ > 400 nm) to detect the trend of the peak of the characteristic signal of the sample with the illumination time.

### Photocatalytic Activity Test

The photocatalytic activity of the as‐obtained photocatalyst was initially studied by selective oxidation of phenylcarbinol (PhCH_2_OH) to benzaldehyde (PhCHO) with visible light irradiation (λ > 400 nm). A 300 W Xenon lamp equipped with a 400 nm cut‐off filter was used as a visible light source (MC‐PF300C, Beijing MerryChange Technology Co., Ltd.). Ten milligrams of catalyst were dispersed in 8 mL of water and 2 mL of benzyl alcohol solution. Before lighting, the system (Xi'an Taikang Technology Co., Ltd.) was evacuated with a mechanical pump to remove air, and then an oxygen bag was attached to the branch port of the reactor. Finally, the suspension was illuminated under visible light. After the reaction, the solution was centrifuged and filtered using a polyether sulfone aluminum membrane with a pore size of 0.22 µm, which was detected by high‐performance liquid chromatography. The apparent quantum efficiency (AQE) for PhCHO production is measured according to Equation (1):

(1) $$\begin{array}{ccc} \mathrm{AQE} & = & 2\times \left(\mathrm{Numbers}\;\mathrm{of}\;\mathrm{PhCHO}\right)/\left(\mathrm{Numbers}\;\mathrm{of}\;\mathrm{incident}\;\mathrm{photons}\right)\\ & & \times \;100\% \end{array}$$

The PhCHO selectivity is calculated with the Equation (2):

(2) $$\mathrm{Selectivity}=\left[C_{\mathrm{PhCHO}}/\left(C_{0}-C_{t}\right)\right]\times 100\%$$

where C_0_, C_t_, and C_PhCHO_ are the concentrations of PhCH_2_OH, the residual PhCH_2_OH and the PhCHO, respectively.

Turnover number (TON) is calculated according to Equation (3):

(3) $$\mathrm{TON}=n\left(\mathrm{PhCHO}\right)/n\left(\mathrm{photocatalysts}\right)$$

where n(PhCHO) and n(photocatalysts) are the amount of substance of PhCHO and photocatalyst, respectively.

Recycling photoactivity tests: After the first photocatalytic reaction, the liquid product and solid catalyst were retained by centrifugation. The solid catalyst was washed with water and ethanol several times repeatedly and then dried under vacuum at 60 °C for the next round of photocatalytic activity test. Because of the possible loss of catalyst during washing, a small amount of fresh catalyst could be added.

## Conflict of Interest

The authors declare no conflict of interest.

## Supporting information

Supporting Information

## Data Availability

The data that support the findings of this study are available from the corresponding author upon reasonable request.
