# Supplementary material for: Metal Sulfide S‐Scheme Homojunction for Photocatalytic Selective Phenylcarbinol Oxidation
Source: Adv Sci (Weinh). 2024 Feb 28;11(17):2400099. doi: 10.1002/advs.202400099 (PMC11077664; doi:10.1002/advs.202400099)
Supplement: Supplementary file 1 — Supporting Information [file ADVS-11-2400099-s001.pdf]

ADVANCED SCIENCE
Open Access

Supporting Information

for *Adv. Sci.*, DOI 10.1002/advs.202400099

Metal Sulfide S-Scheme Homojunction for Photocatalytic Selective Phenylcarbinol Oxidation

Huijun Zhang, Yujie Gao, Sugang Meng, Zengrong Wang, Peixian Wang, Zhongliao Wang, Chengwei Qiu, Shifu Chen, Bo Weng* and Yu-Ming Zheng*

Supporting Information

Metal Sulfide S-Scheme Homojunction for Photocatalytic Selective Phenylcarbinol Oxidation

Huijun Zhang#, Yujie Gao#, Sugang Meng*, Zengrong Wang, Peixian Wang, Zhongliao Wang, Chengwei Qiu, Shifu Chen, Bo Weng*, Yu-Ming Zheng

H. Zhang, Dr. Z. Wang, Prof. S. Chen, Prof. S. Meng
Key Laboratory of Green and Precise Synthetic Chemistry and Applications, Ministry of Education, College of Chemistry and Materials Science, Huaibei Normal University, Huaibei 235000, P. R. China.
E-mail: sgmeng@chnu.edu.cn.
Prof. B. Weng, Prof. Y. Zheng
CAS Key Laboratory of Urban Pollutant Conversion, Institute of Urban Environment, Chinese Academy of Sciences, 1799 Jimei Road, Xiamen 361021, China.
University of Chinese Academy of Sciences,19A Yuquan Road, Beijing 100049, China.
E-mail: bweng@iue.ac.cn
Y. Gao, Prof. B. Weng
cMACS, Department of Microbial and Molecular Systems, KU Leuven, Celestijnenlaan 200F, 3001 Leuven, Belgium.
Dr. Z. Wang, Prof. S. Meng
High Field Magnetic Laboratory, Hefei Institutes of Physical Science, Chinese Academy of Sciences, Hefei 230031, P. R. China.
Dr. P. Wang, Prof. S. Meng
School of Chemistry and Chemical Engineering/State Key Laboratory Incubation Base for Green Processing of Chemical Engineering, Shihezi University.
Shihezi 832003, P. R. China.
Mr. C. Qiu
State Key Lab of Photocatalysis on Energy and Environment, College of Chemistry, Fuzhou University, Fuzhou 350116, China

# These authors contributed equally to this work.

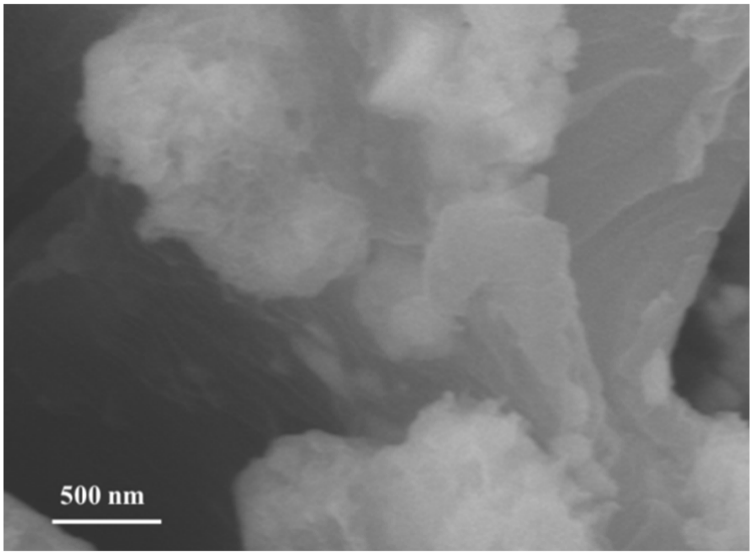

Figure S1. SEM image of n-CIS.

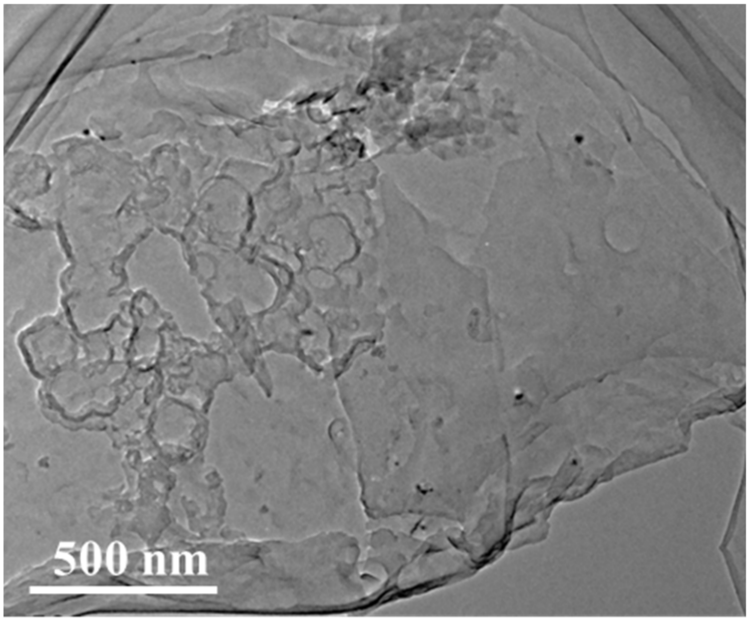

Figure S2. TEM image of n-CIS.

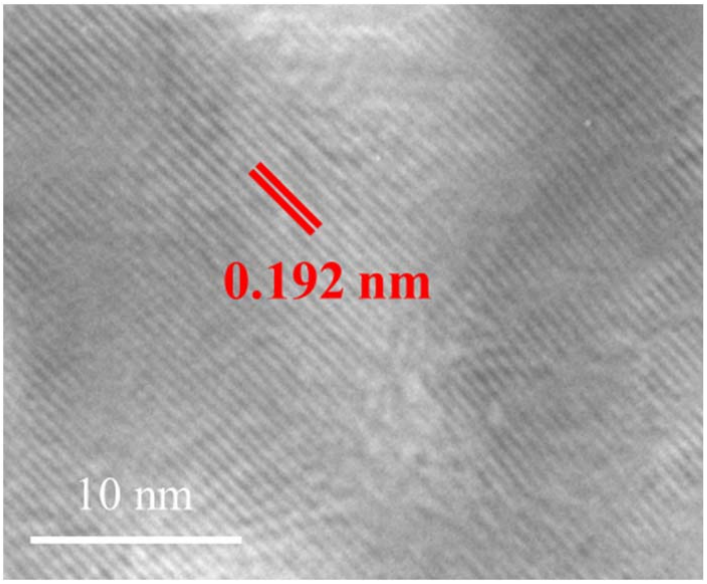

Figure S3. HRTEM image of n-CIS.

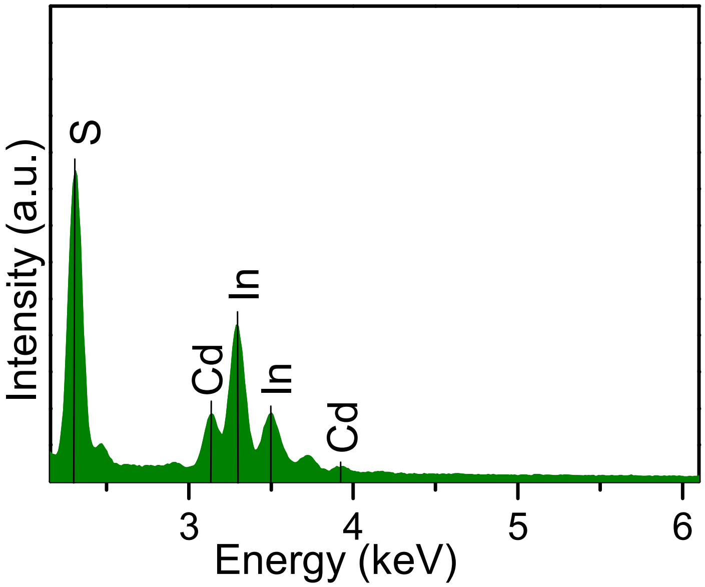

Figure S4. EDX spectrum of n-CIS.

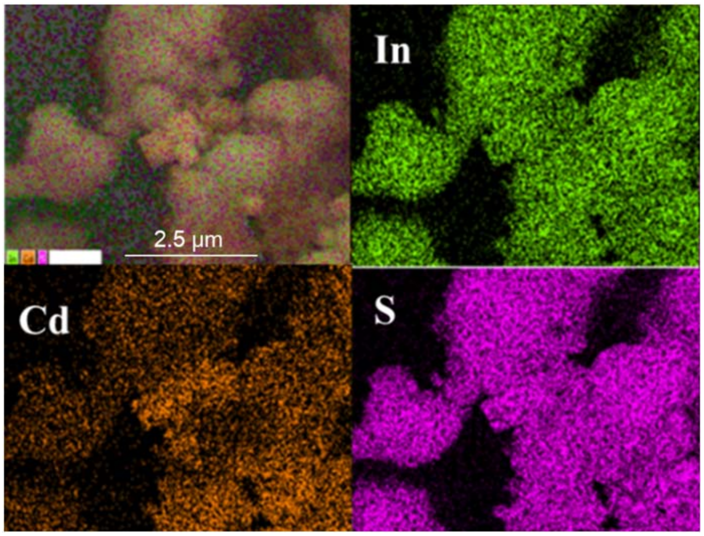

Figure S5. EDX mapping images of n-CIS.

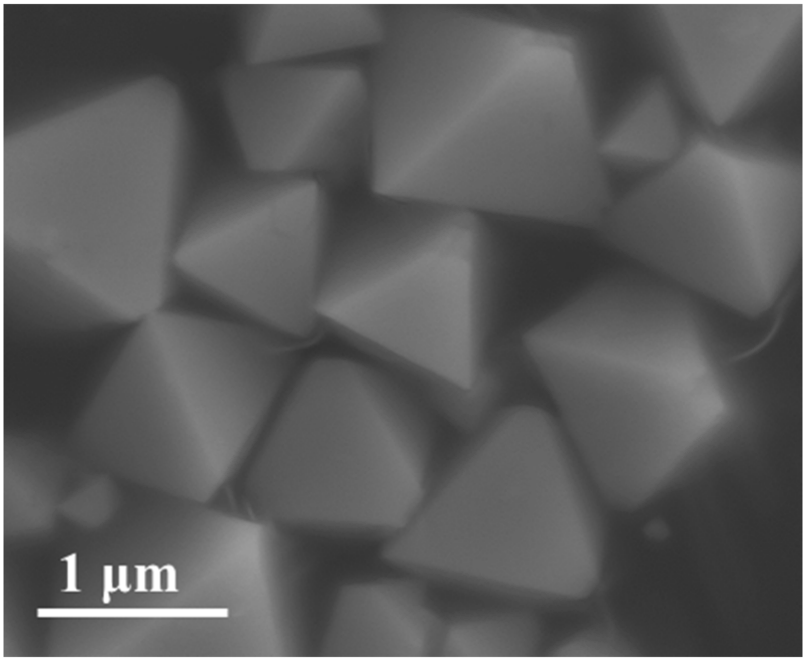

Figure S6. SEM image of o-CIS.

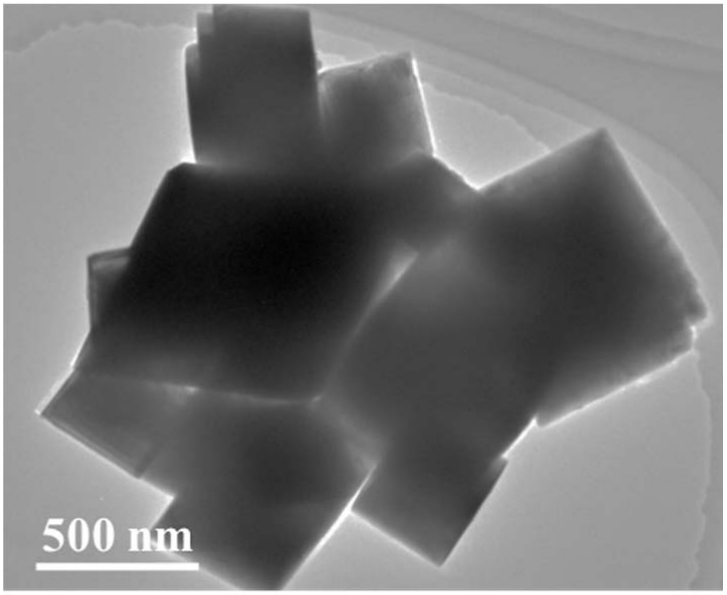

Figure S7. TEM image of o-CIS.

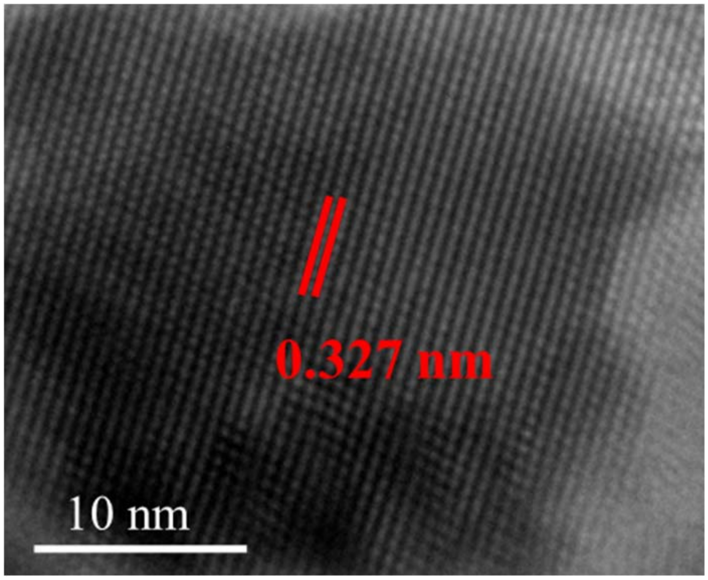

Figure S8. HRTEM image of o-CIS.

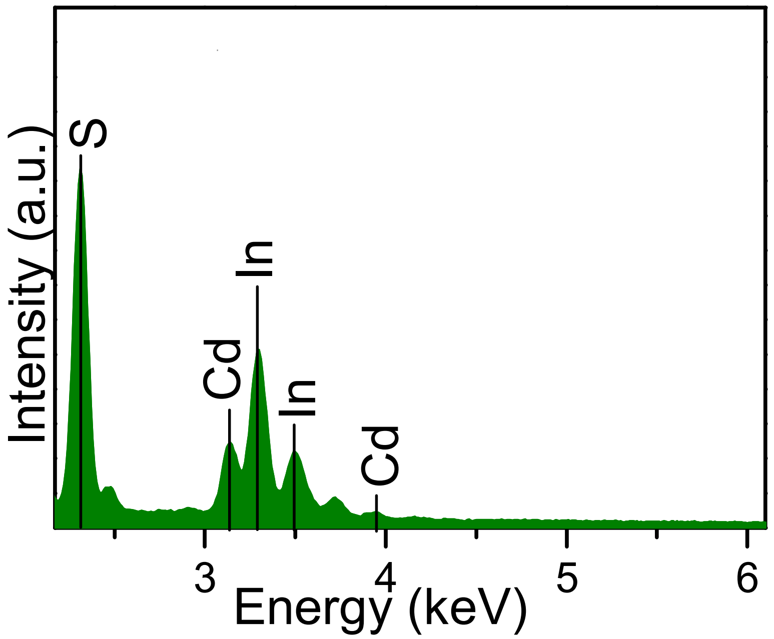

Figure S9. EDX spectrum of o-CIS.

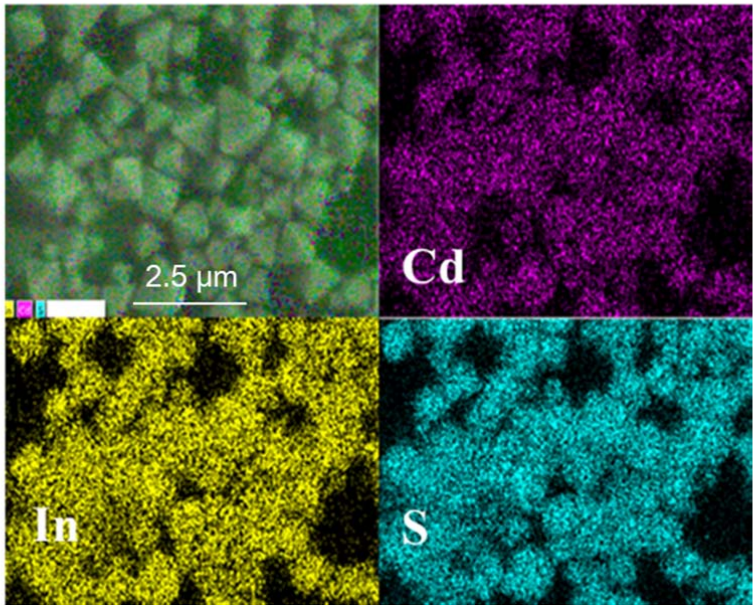

Figure S10. EDX mapping images of o-CIS.

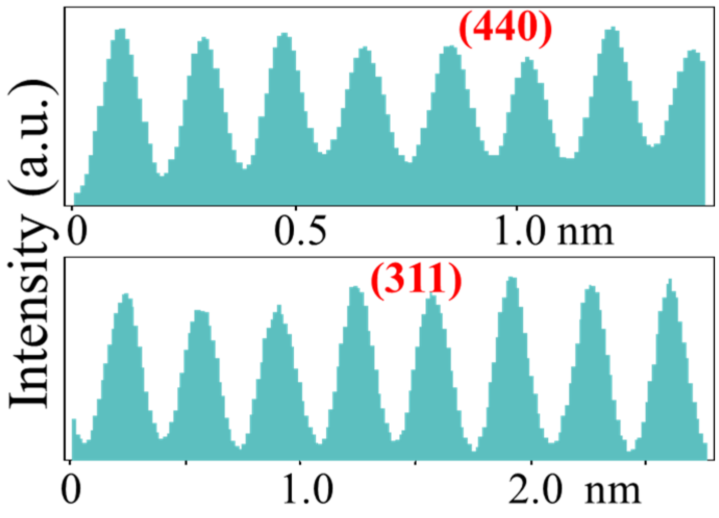

Figure S11. The FFT corresponding to HRTEM of n-CIS/o-CIS homojunction.

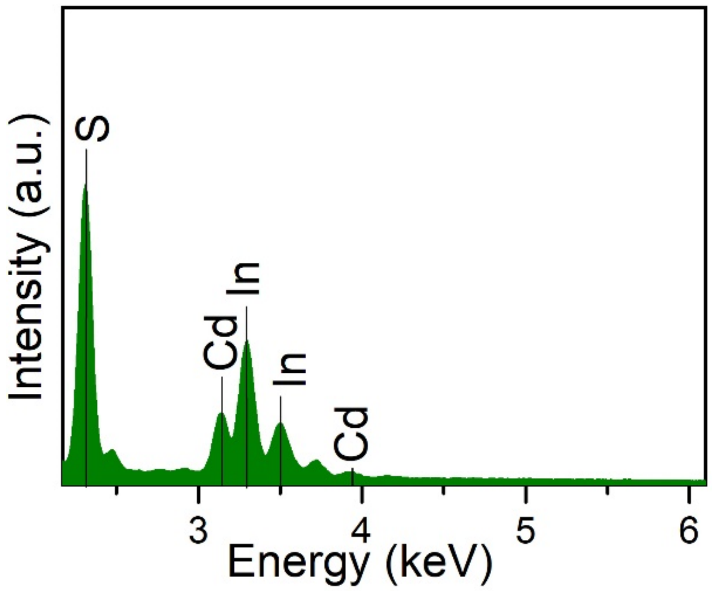

Figure S12. EDX spectrum of n-CIS/o-CIS homojunction.

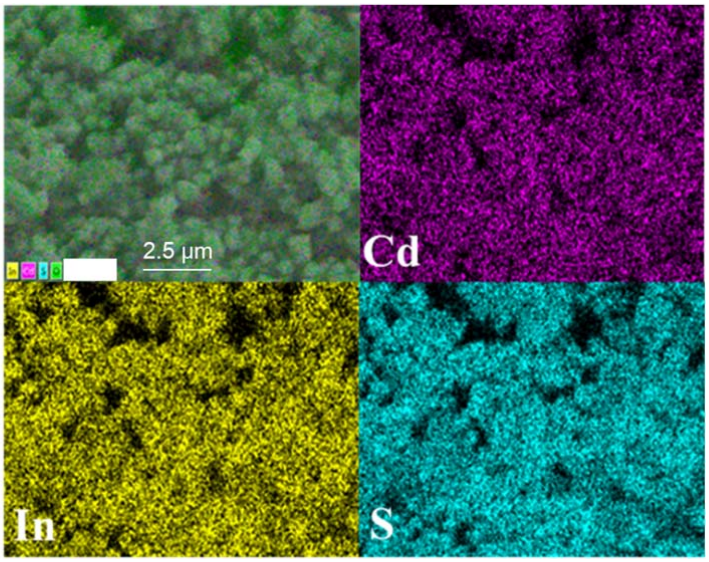

Figure S13. Element mapping images of n-CIS/o-CIS homojunction.

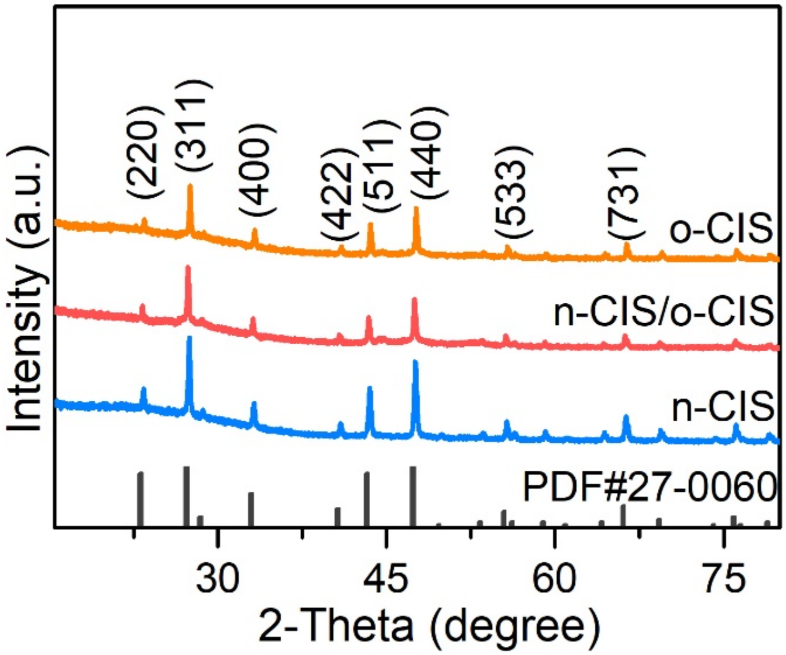

Figure S14. XRD patterns of n-CIS, o-CIS and n-CIS/o-CIS homojunction.

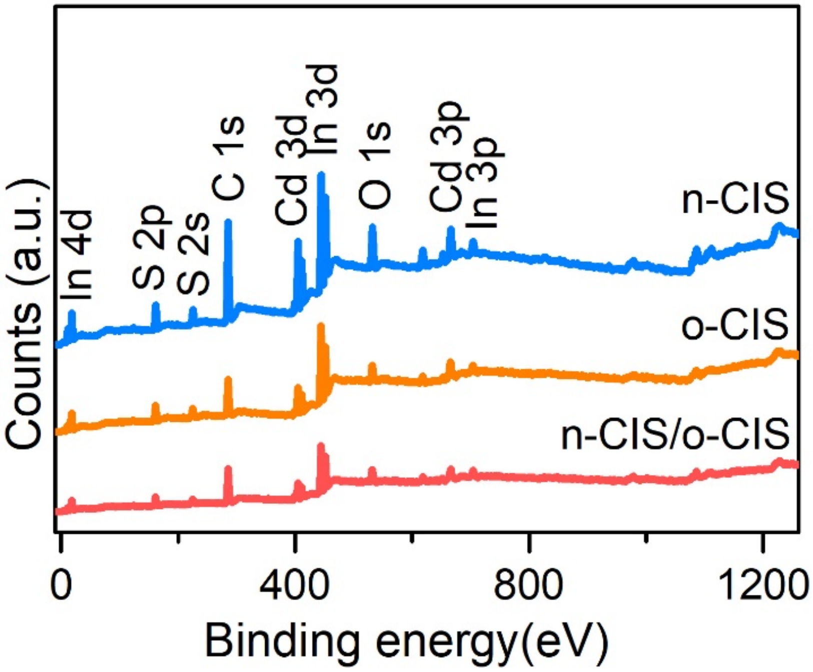

Figure S15. XPS survey spectra of n-CIS, o-CIS and n-CIS/o-CIS homojunction.

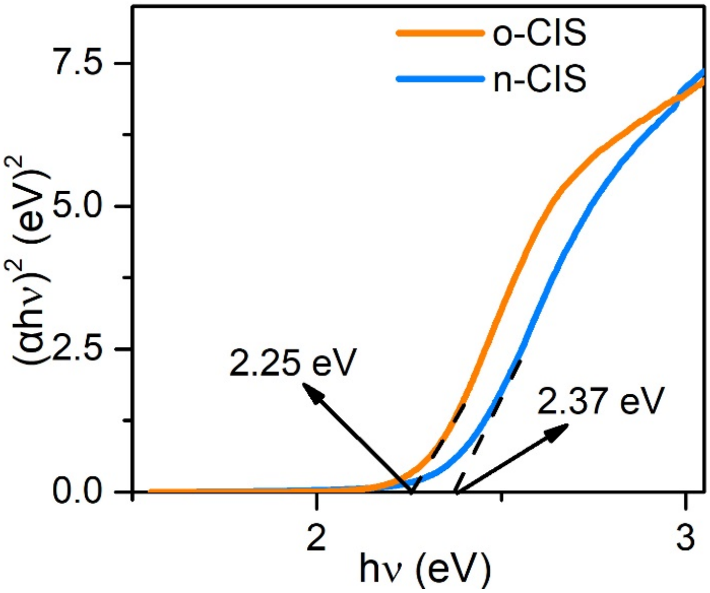

Figure S16. The band-gap energies of n-CIS and o-CIS.

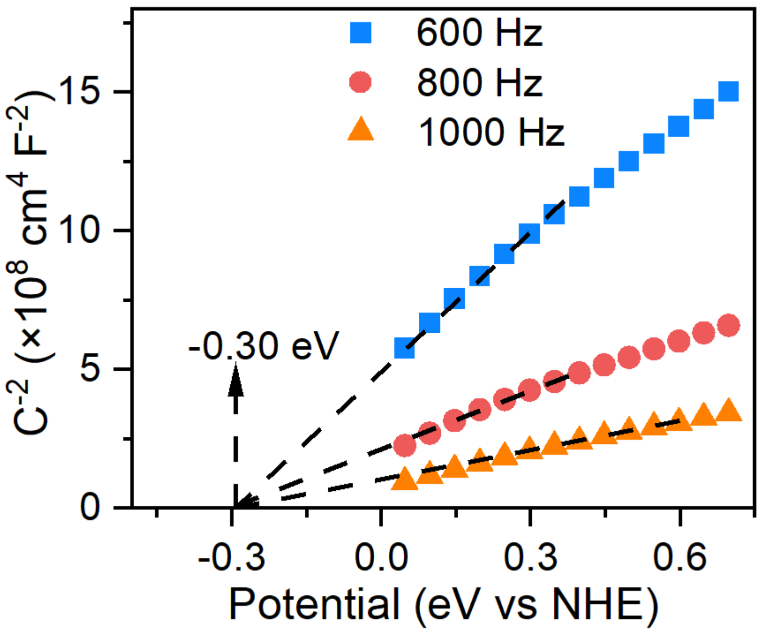

Figure S17. Mott–Schottky plots for n-CIS.

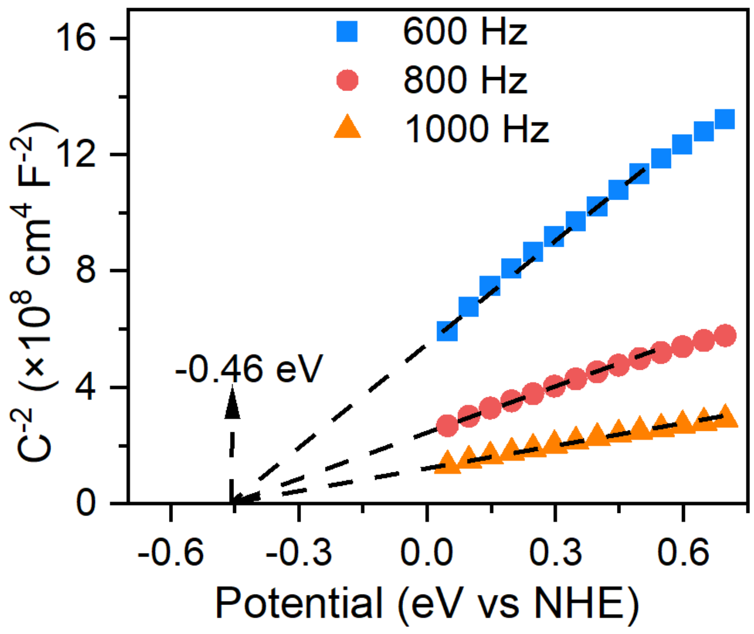

Figure S18. Mott–Schottky plots for o-CIS.

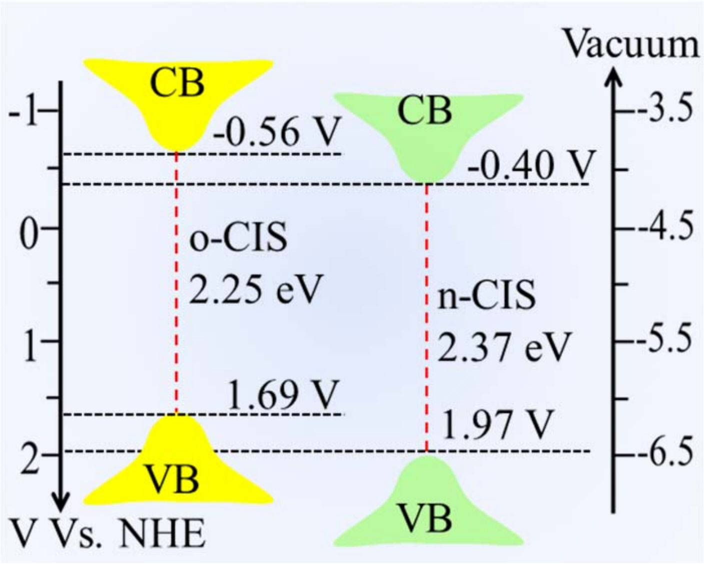

Figure S19. The band energies of n-CIS and o-CIS.

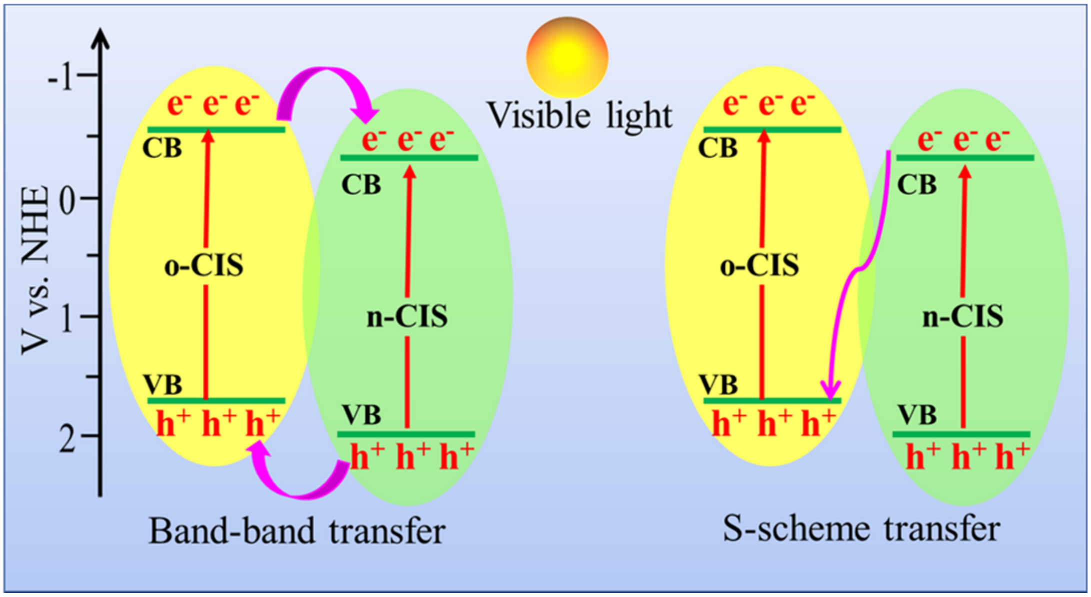

Figure S20. The possible mechanism of photoexcited charge separation and transportation over n-CIS/o-CIS homojunction under visible light.

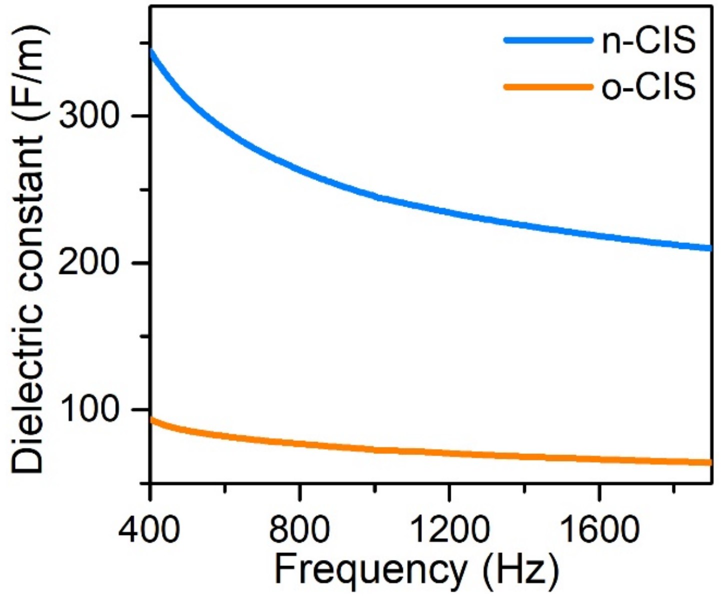

Figure S21. The dielectric constants of n-CIS and o-CIS.

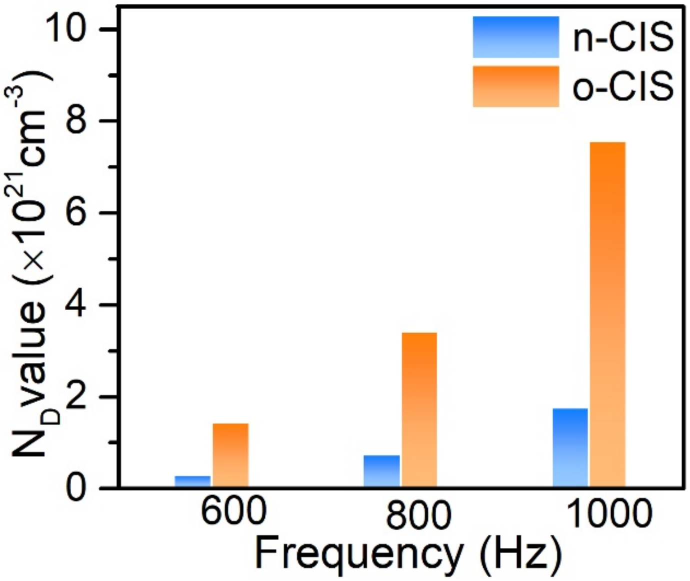

Figure S22. Comparison of carrier concentrations of n-CIS and o-CIS at different frequencies.

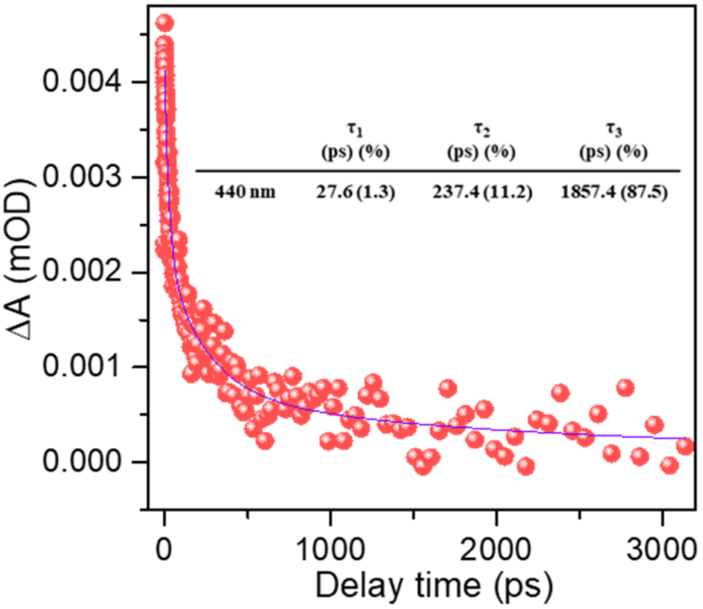

Figure S23. Normalized decay kinetic curves at 440 nm inn-CIS/o-CIS homojunction.

Note: The photogenerated electrons are easily captured by the electron trapping states to become the shallowly trapped electrons, leading to a relatively short lifetime (τ_1= 27.6 ps). Since the hole-trapping state resides above the valence band (VB), the second electron decay pathway (τ_2 = 237.4 ps) is attributed to the recombination of photogenerated electrons with trapped holes. The slowest electron quenching process is the recombination of electrons in the CB and holes in VB (τ_3 =1857.4 ps), aligning with the estimated lifetime in the TRPL results.

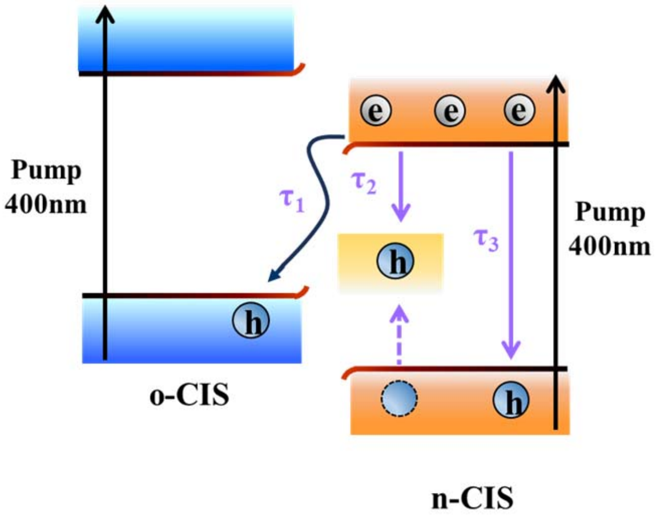

Figure S24. Schematics for the decay pathways of photogenerated electrons in n-CIS/o-CIS homojunction.

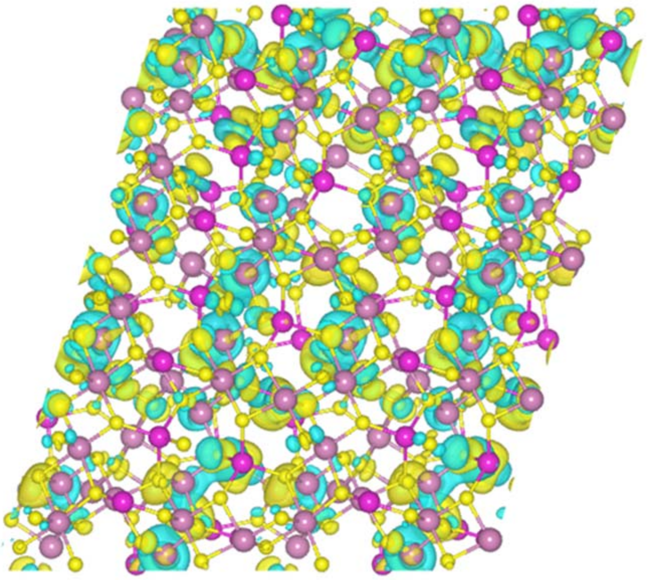

Figure S25. Simulated differential charge density distribution at the interface between n-CIS and o-CIS with an isosurface of 1.5*10^{-3} e/$Å^3$: top view.

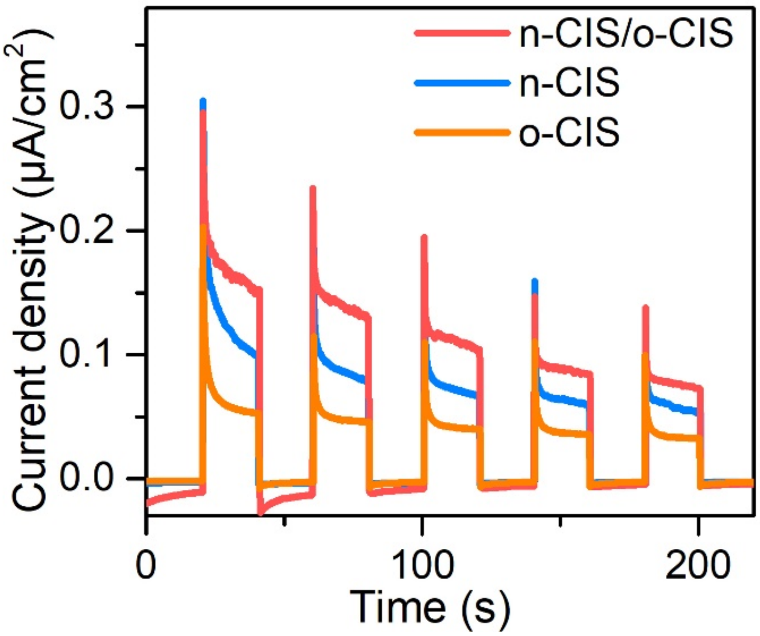

Figure S26. Photocurrent response of the n-CIS, o-CIS and n-CIS/o-CIS samples under visible light irradiation.

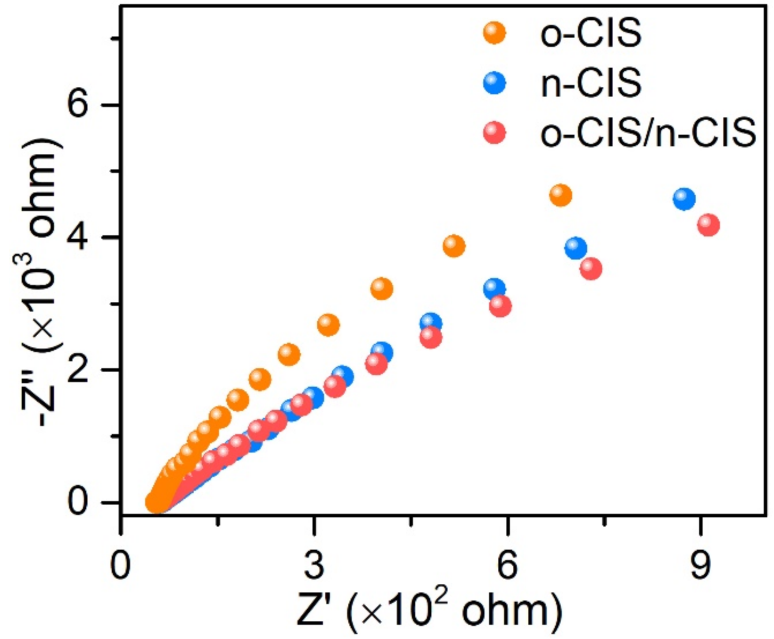

Figure S27. EIS Nyquist plots of the n-CIS, o-CIS and n-CIS/o-CIS samples.

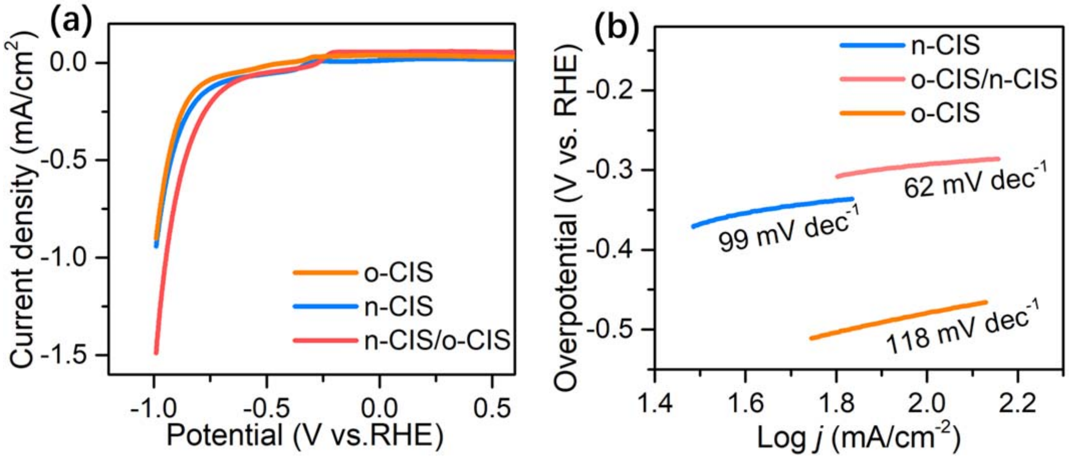

Figure S28. (a) Visible-light-driven LSV curves and (b) corresponding Tafel slopes of n-CIS, o-CIS and n-CIS/o-CIS homojunction.

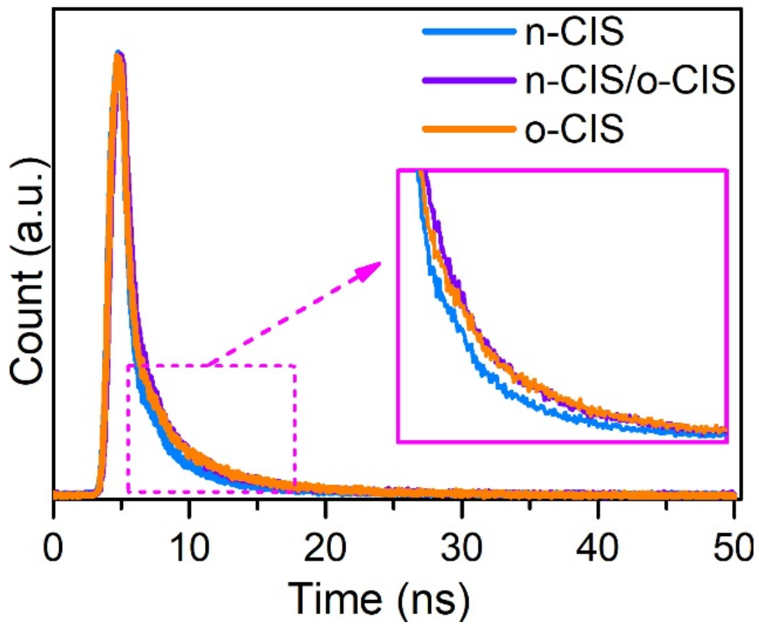

Figure S29. TR-PL spectra of the n-CIS, o-CIS and n-CIS/o-CIS samples.

Table S1. The lifetime of charge carriers calculated from TR-PL spectra.

Sample	A_1(%)	τ_1 (ns)	A_2(%)	τ_2 (ns)	τ_a (ns)
n-CIS	0.23	4.253	99.77	0.830	0.869
n-CIS/o-CIS	99.29	1.012	0.71	4.786	1.135
o-CIS	0.55	5.504	99.45	0.994	1.129

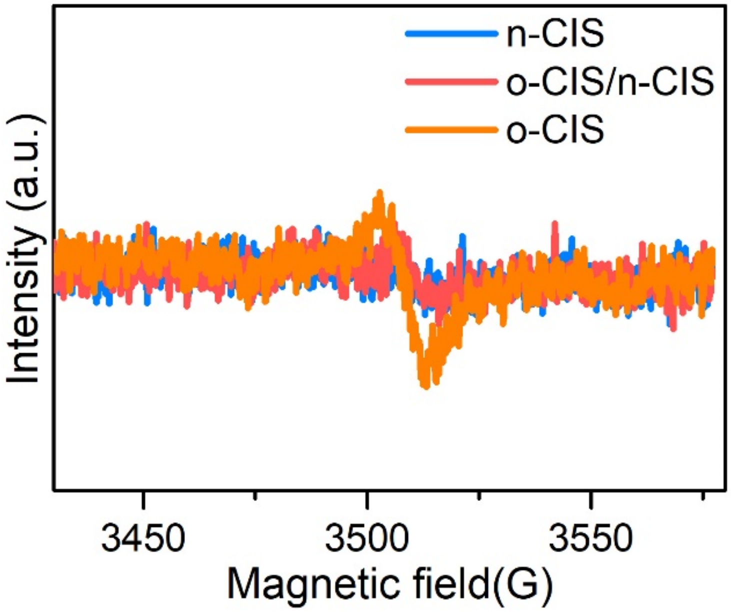

Figure S30. EPR spectra of n-CIS, o-CIS and n-CIS/o-CIS samples.

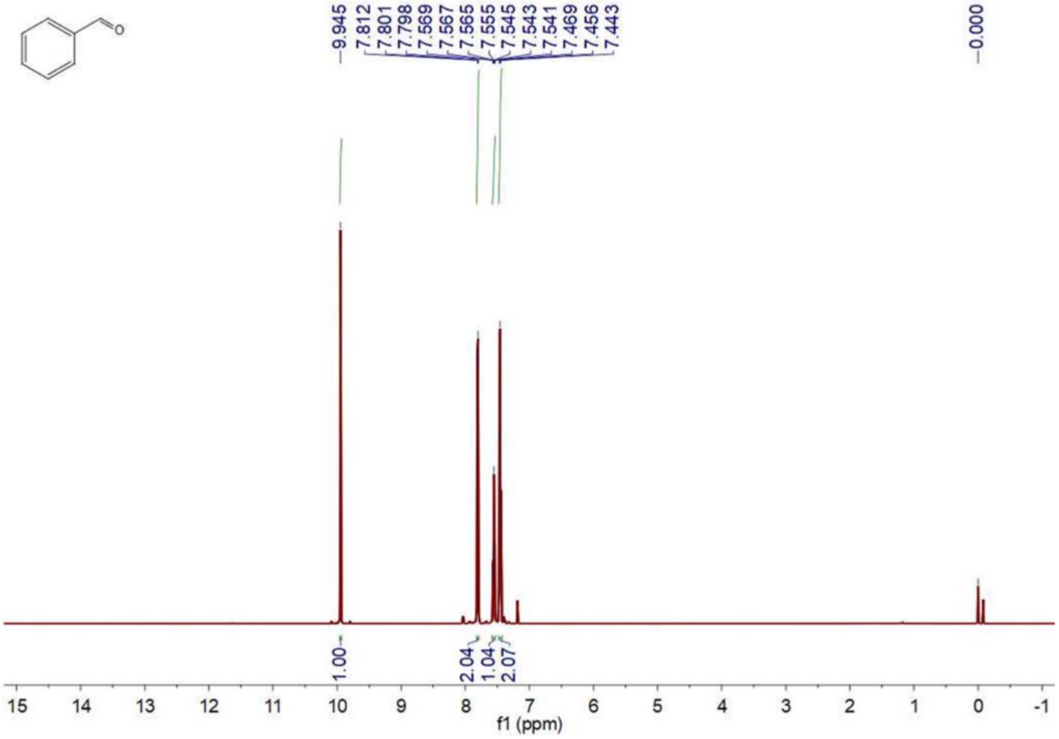

Figure S31. ^{1}H NMR spectrum of benzaldehyde (^{1}H NMR (600 MHz, $CDCl_3$) δ 9.95 (s, 1H), 7.82 – 7.79 (m, 2H), 7.58 – 7.53 (m, 1H), 7.46 (t, *J* = 7.7 Hz, 2H).) .

Table S2. Comparison of photocatalytic activity of $CdIn_2S_4$ (CIS) materials for selective oxidation of $PhCH_2OH$.

Catalysts	Light source	PhCHO (mmol g^{-1} h^{-1})	PhCHO selectivity (%)	Ref.
CIS	300 W Xe lamp ($\lambda > 420$ nm)	7.4	98.8	[1]
CdS/CIS	300 W Xe lamp ($\lambda > 420$ nm)	3.3	80.9	[2]
CIS	300 W Xe lamp ($\lambda > 420$ nm)	7.5	99.0	[3]
n-CIS/o-CIS	300 W Xe lamp ($\lambda > 400$ nm)	19.9	99.9	This work

[1] X. J. Ye, Y. H. Chen, C. C. Ling, J. F. Zhang, S. G. Meng, X. L. Fu, X. C. Wang, S. F. Chen. *Chem. Eng. J.* **2018**, *348,* 966-977.

[2] Q. Q. Zhang, J. X. Wang, X. J. Ye, Z. Z. Hui, L. Q. Ye, X. C. Wang, S. F. Chen. *ACS Appl. Mater. & Inter.* **2019**, *11,* 46735-46745.

[3] C. C. Ling, X. J. Ye, J. H. Zhang, J. F. Zhang, S. J. Zhang, S. G. Meng, X. L. Fu, S. F. Chen. *Sci. Rep.* **2017**, *7*, 1-16.

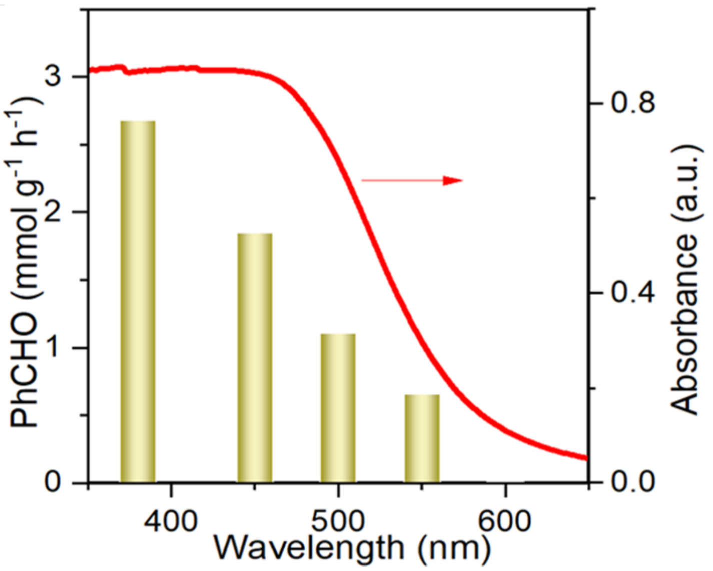

Figure S32. The activity of the n-CIS/o-CIS sample under different incident lights.

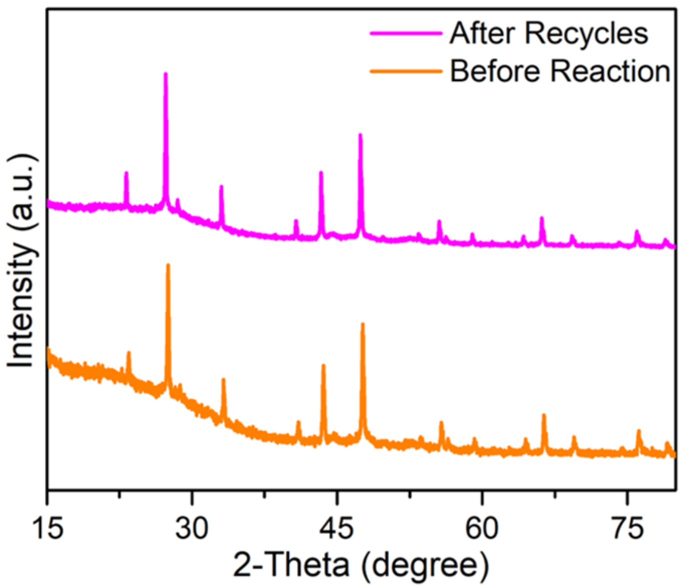

Figure S33. XRD patterns of the n-CIS/o-CIS samples before and after the photocatalytic reaction.

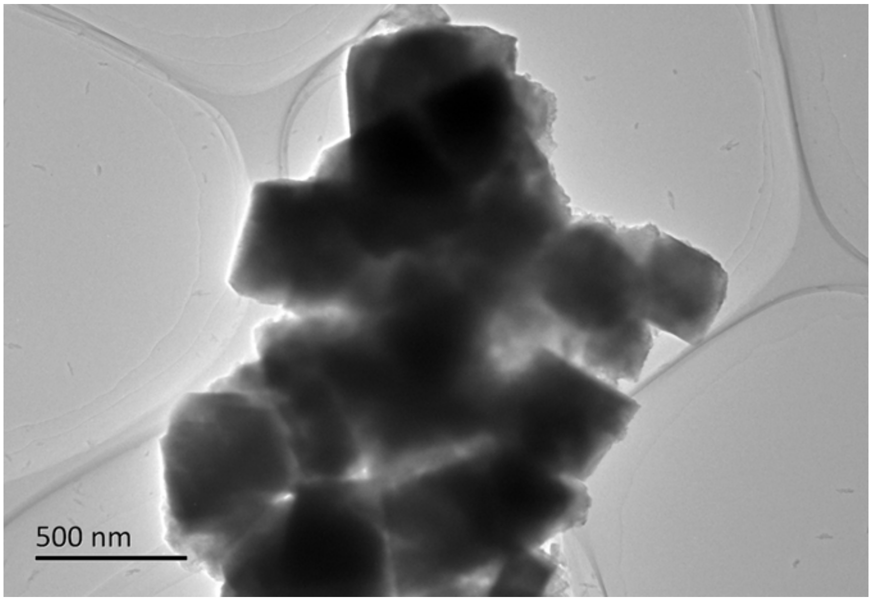

Figure S34. TEM image of the n-CIS/o-CIS sample after the photocatalytic reaction.

Table S3. Photocatalytic activity of the n-CIS/o-CIS sample for selective oxidation of various aromatic alcohols into corresponding aldehydes under visible light irradiation.

Entry	Substrate	Product	Yield (mmol g^{-1} h^{-1})	Selectivity (%)
1	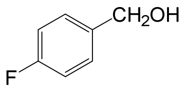	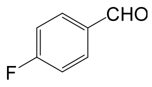	11.6	99.6
2	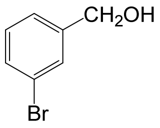	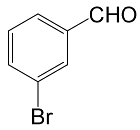	9.8	99.0
3	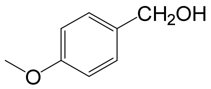	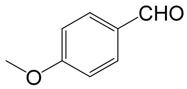	11.3	99.5
4	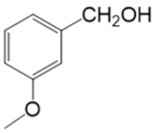	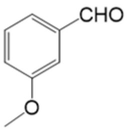	10.7	99.3
5[a]	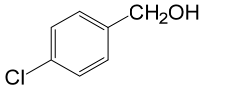	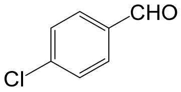	5.1	99.5
6[a]	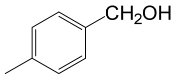	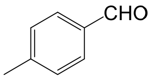	6.1	99.2

[a] 4-chlorobenzyl alcohol and 4-methylbenzyl alcohol are solid reagents and insoluble in water, and the selected solvent is acetonitrile.

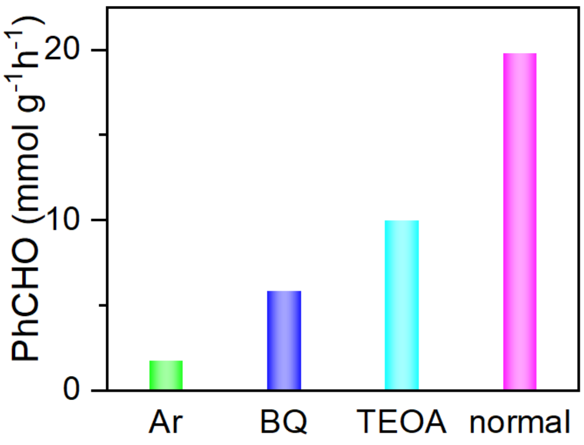

Figure S35. Control experiments for photocatalytic phenylcarbinol oxidation over the n-CIS/o-CIS (normal, no scavenger added; BQ and TEOA are scavengers for superoxide radicals and holes, respectively).

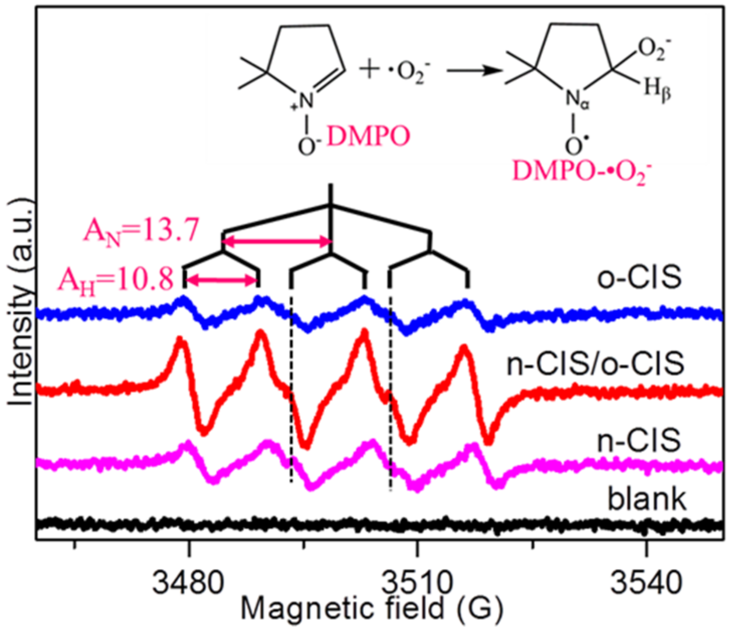

Figure S36. EPR spectra of DMPO-•O_2^- over n-CIS, o-CIS and n-CIS/o-CIS homojunction under visible light irradiation.

Note: The characteristic peak of DMPO-•O_2^- (A_N = 13.7, A_H = 10.8) can be clearly observed under visible light irradiation, and the EPR peak intensity of n-CIS/o-CIS is significantly stronger than that of n-CIS and o-CIS, which also proves that the •O_2^- radical produced by n-CIS/o-CIS is significantly more than that of n-CIS and o-CIS.

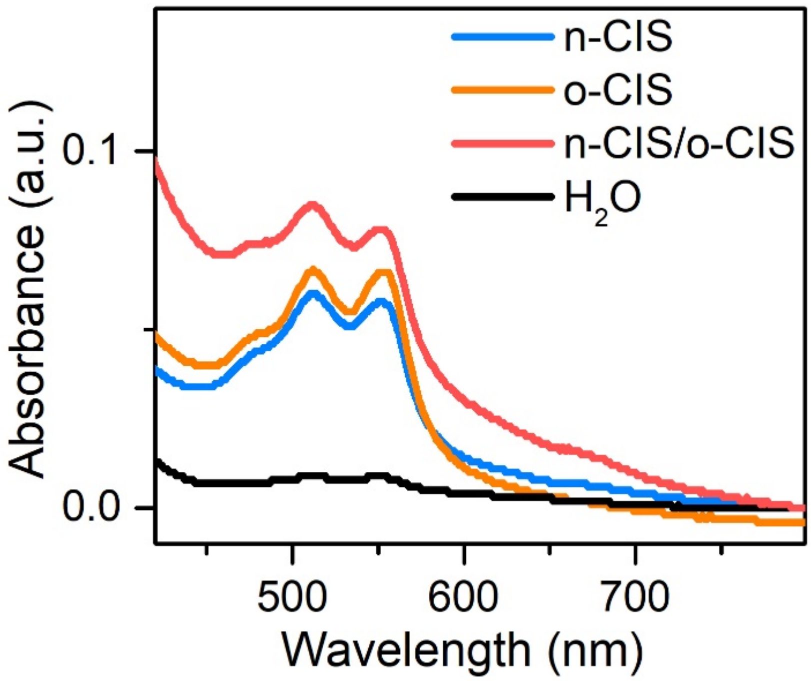

Figure S37. UV–Vis spectra of hydrogen peroxide production over different photocatalysts.

Note: Detection of H_2O_2 was performed using the DPD/POD method. The principle is as follows: H_2O_2 can oxidize peroxidase product from horseradish (POD), and the oxidation products of POD will then oxidize N, N-diethyl-1,4-phenyldiammonium sulfate (DPD) to the positive ion DPD$\bullet^+$. DPD$\bullet^+$ is a pink compound with two characteristic absorption peaks at 510 nm and 551 nm. The experimental procedure is shown below. Detailly, 0.1 g DPD was dissolved in 10 mL 0.1 M H_2SO_4 solution, and 10 mg POD was dissolved in 10 mL H_2O. The DPD solution and POD solution should be freshly prepared and stored at 2-3 °C in the dark. 10 μL DPD and 10 μL POD solution were successive added into 3 mL of the reaction solution. After the solution was mixed evenly, the solution was detected by using a UV-visible diffuse reflectance spectrophotometer. As shown in **Figure S37**, two characteristic absorption peaks (510 nm and 551 nm) belonged to H_2O_2 could be observed over n-CIS, o-CIS and n-CIS/o-CIS, indicating that H_2O_2 was generated in the process of photocatalytic oxidation of benzyl alcohol to benzaldehyde over the as-prepared photocatalysts under visible light irradiation.

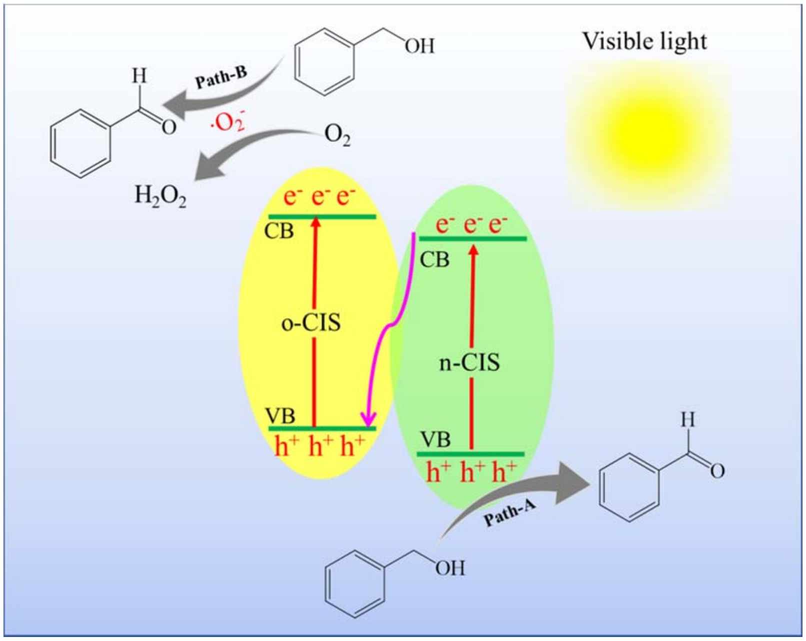

Figure S38. Reaction pathways of benzyl alcohol oxidation over n-CIS/o-CIS catalysts.

Note: Figure S38 presents a rational process for the selective oxidation of $PHCH_2OH$ to produce PHCHO over n-CIS/o-CIS catalysts. The S-scheme homojunction interface formed between n-CIS and o-CIS in the n-CIS/o-CIS composites resulted in stronger charge interactions and the formation of the internal electric field. Upon excitation by visible light irradiation, photogenerated hole-electron pairs are first formed. Driven by the internal electric field and energy band bending, the photoexcited electrons in the CB of the n-CIS and the photoexcited holes in the VB of the o-CIS are combined. Notably, the photogenerated holes with strong oxidation ability and the electrons with high reduction ability retain on the n-CIS side and the o-CIS surface, respectively. Therefore, the adsorbed $PhCH_2OH$ reactants on the surface of n-CIS could be directly converted into PhCHO products by holes (Path-A). The photogenerated electrons on the o-CIS surface can reduce the adsorbed O_2 molecules to obtain reactive oxygen species ($•O_2^-$). $•O_2^-$ also oxidizes $PHCH_2OH$ to the target product of PHCHO (Path-B), along with the production of H_2O_2 as a by-product (H_2O_2 was detected (**Figure S37**, Supporting Information)).
